# Mechanism of Silybin in Alleviating Liver Damage Induced by Heat Stress of Peking Ducks

**DOI:** 10.3390/ani16162615

**Published:** 2026-08-20

**Authors:** Ziyue Zhang, Shihao Xuan, Junfeng Lv, Zhaofei Xia, Dong Zhang, Jing Chen, Zouran Lan, Guisheng Wang, Yanhan Liu

**Affiliations:** 1 Shandong Vocational Animal Science and Veterinary College, Weifang 261061, China; zzyue0211@126.com; 2 College of Veterinary Medicine, China Agricultural University, Beijing 100193, China; zhaofeixiacau@163.com; 3 College of Veterinary Medicine, Shandong Agricultural University, Taian 271018, China; lixuanshihao@163.com; 4 Poultry Institute, Shandong Academy of Agricultural Sciences, Jinan 250100, China; nkyljf@163.com; 5 Shandong Provincial Center for Animal Disease Control, Jinan 250100, China; zhangdong_jn@163.com (D.Z.); chenjing@shandong.cn (J.C.); lanzr@163.com (Z.L.)

**Keywords:** silybin, peking ducks, heat stress, liver, transcriptome

## Abstract

This study aimed to explore the protective mechanism of silybin against heat stress-induced liver injury in Peking ducks as a result of oxidative stress, inflammation, and lipid metabolism. One hundred and ninety-five 1-day-old healthy male Peking ducks with similar body weights were randomly divided into a control group, a heat-stress group, and three intervention groups treated with 400, 800, and 1600 mg/kg silybin under heat-stress from 21 d to 35 d for two weeks. The function indices, oxidative stress, inflammatory factor levels, histopathological changes and non-targeted lipidomic profiling in liver as well as growth performance were evaluated. Results showed that silybin supplementation partially alleviated the heat-stress-induced decreases in average daily feed intake and body weight gain (*p* > 0.05). Silybin supplementation at 400 and 800 mg/kg significantly reduced the heat-stress-induced increases in alanine aminotransferase (ALT), aspartate aminotransferase (AST), and γ-glutamyl transpeptidase (γ-GGT) (*p* < 0.05), whereas the effect of 1600 mg/kg silybin was not significant. Albumin (ALB) and globulin (GLB) levels were mainly improved in the 800 mg/kg group, and GLB was also increased in the 400 mg/kg group (*p* < 0.05). Silybin supplementation significantly improved hepatic antioxidant capacity, especially in the 400 and 800 mg/kg groups, as indicated by increased superoxide dismutase (SOD) activity and total antioxidant capacity (T-AOC), and decreased malondialdehyde (MDA) and reactive oxygen species (ROS) levels (*p* < 0.05). In addition, the 400 and 800 mg/kg groups showed more significant reductions in the pro-inflammatory factors IL-6 and TNF-α, while IL-10 was increased in silybin-supplemented groups (*p* < 0.05). Lipid metabolism disorders were improved through regulation of the sphingolipid and glycerophospholipid metabolism pathways, while it alleviated heat stress damage by reducing the expression of heat shock proteins. In conclusion, silybin holds promise as an effective feed additive to alleviate heat stress in poultry. This study provides a theoretical basis and practical approach for improving the health and productive performance of Peking ducks under heat-stress conditions.

## Simple Summary

Heat stress can reduce duck growth and damage the liver through oxidative stress, inflammation, and disturbed lipid metabolism. This study evaluated whether dietary silybin could protect Peking ducks exposed to heat stress. Silybin supplementation partially alleviated heat-stress-induced growth depression and reduced several markers of liver injury, strengthened antioxidant capacity, moderated inflammatory responses, and lessened histological liver damage and lipid accumulation. Lipidomic analysis further indicated that silybin affected sphingolipid and glycerophospholipid metabolism. These results support the use of silybin as a promising feed additive to alleviate adverse impacts of heat stress and improve poultry health.

## 1. Introduction

In current intensive and large-scale livestock production, many farms face inadequate or insufficient ventilation equipment. This, coupled with global warming, has resulted in heat stress becoming a major obstacle to the industry [1]. In addition, poultry lack sweat glands and can only rely on behavioral adaptations such as panting, spreading their wings, and drinking plenty of water to dissipate body heat. However, these mechanisms are often insufficient, and birds remain vulnerable to heat stress. Studies have shown that heat stress reduces poultry production performance indicators such as weight and feed intake while triggering a series of physiological and metabolic disorders, including damaging the integrity of the intestinal barrier and gut microbial balance, interfering with normal fat metabolism, reducing reproductive efficiency, and weakening the immune response [2]. At the molecular level, heat stress causes metabolic disorders and an increase in the production of inflammatory factors and reactive oxygen species, damaging the body’s antioxidant capacity and resulting in oxidative stress [3,4]. Furthermore, heat stress can damage tissues and organs and disrupt normal liver tissue structure and function.

With the intensification of global warming, heat stress resulting from elevated environmental temperatures is becoming increasingly prevalent and has gradually become an environmental factor resulting in economic losses in the livestock and poultry industries. Their lack of sweat glands renders poultry extremely vulnerable to heat stress, resulting in intuitive physiological abnormalities and reduced production performance, as well as systemic inflammatory responses, circulatory disorders, organ failure, and other pathological changes [5,6,7,8]. In recent years, an increasing number of researchers have focused on heat stress, but the underlying mechanisms in Peking ducks have not yet been fully elucidated. There is still a lack of robust and effective mitigation strategies. Many studies have shown that heat stress can reduce the activity of antioxidant enzymes, increase the expression levels of inflammatory factors such as TNF-α and IL-1β, and disrupt body homeostasis [9,10]. Research indicates that heat stress can also cause liver injury by inducing inflammation and oxidative stress [11,12,13]. Silybin is an excellent anti-inflammatory and antioxidant in the liver, and thus a strong candidate agent for alleviating heat stress.

Metabolomics using high-throughput mass spectrometry can quantitatively and qualitatively analyze metabolites and their in vivo environmental interactions. It enables a holistic and systematic analysis of small-molecule metabolites within organisms, offering researchers a more intuitive understanding of metabolic processes at the single-cell or even organelle level. This provides new perspectives for investigating the relationship between disease development and the metabolism of bioactive substances, while paving the way for new research directions [14].

Silybin is hepatoprotective, antioxidative, anti-inflammatory, and anti-tumor. Studies have shown that it not only alleviates liver damage by decreasing the expression of inflammatory factors, inhibiting nuclear factor kappa B (NF-κB) signal transduction, and regulating NOD-like receptor thermal protein domain associated protein 3 (NLRP3), but also enhances antioxidant capacity by eliminating free radicals and increasing the activity of antioxidant enzymes, thereby improving the health status of organisms. Nevertheless, whether silybin has a protective effect against heat-stress-induced liver injury in Peking ducks remains unreported. As the metabolic hub of the body, the liver plays an irreplaceable role in regulating the metabolism of numerous biomolecules, such as sugar, protein, lipids, and microorganisms. Furthermore, the rate and extent of lipid synthesis and accumulation in the body are closely linked to liver health. However, the liver is highly susceptible to external variables such as diet, disease, and environmental stressors. Heat stress-induced liver damage leads to systemic metabolic disorders, and studies have found that it can upregulate genes related to lipogenesis and increase the levels of very-low-density lipoprotein (VLDL) and fatty acid synthase (FASN) involved in fat synthesis and transport. This results in accelerated lipogenesis and transport, increased liver burden, and disrupted normal lipid metabolism [15]. Thus, elucidation of the relationship between metabolites or metabolic pathways and physiological changes to gain a deeper understanding of the underlying biological mechanisms is of great significance for comprehensively recognizing and managing disease, as well as for developing rational intervention strategies. This study aimed to investigate the protective mechanism of silybin against heat-stress-induced liver injury in Peking ducks by establishing a heat-stress model and employing non-targeted metabolomics technology. Findings are expected to provide a theoretical basis for the administration of silybin as an effective agent to alleviate heat-stress-induced liver injury in poultry and offer new insights into solving practical production problems in the duck production industry.

## 2. Materials and Methods

### 2.1. Experimental Animals and Diets

The silybin (purity≥98%) used in this study was purchased from Shaanxi Haokang Biotechnology Co., Ltd., Xi’an, China. Peking ducks were 1-day-old healthy male Jingdian Peking ducks. The corn-soybean meal basal diet was formulated in accordance with the standard < Peking Duck Part 1: Technical specification for farming commercial duck > (DB11/T 012.1—2023) [16], and the composition and nutritional information of the basal diet are detailed in Table 1.

### 2.2. Experimental Design and Feeding Management

All experimental procedures followed the regulations of the Animal Welfare and Animal Experiment Ethics Committee of China Agricultural University (Approval number AW61303202-2-3, Date 16 March 2023). All animal experiments were carried out at the Nankou Pilot Base of the Chinese Academy of Agricultural Sciences.

A total of 195 1-day-old healthy male Peking ducks with similar body weights (50.12 ± 0.06 g) were randomly selected and divided into five groups: a control group (C), a heat-stress group (HS), a low-dose + heat-stress group (L + HS), a middle-dose + heat-stress group (M + HS), and a high-dose + heat-stress group (H + HS). Each group consisted of three independent replicates of 13 ducks each. From 1 to 35 d, the C and HS groups were fed with the basal diet, while the L + HS, M + HS, and H + HS groups were fed with a basal diet supplemented with 400 mg/kg, 800 mg/kg, and 1600 mg/kg silybin, respectively. From 21 to 35 d, ducks in the HS, L + HS, M + HS, and H + HS groups were exposed to heat stress in a temperature-controlled room maintained at 32 ± 2 °C and 55 ± 5% relative humidity from 9:00 to 18:00 each day, while ducks in the C group were maintained under normal temperature conditions.

### 2.3. Behavioral Observation and Measurement

During the two-week experimental period, the body weights, feed intake, and water consumption of all ducks in each group were recorded along with their perceived mental status and any abnormal behavior.

### 2.4. Sample Collection and Processing

After the heat stress period, one duck (35 d of age) was randomly selected from each replicate of each group (C group, HS group, L + HS group, M + HS group, and H + HS group) for blood and liver sampling, with three biological replicates per group. Blood was collected (5 mL) from the jugular vein for serum liver function indicators, including total protein (TP), alanine aminotransferase (ALT), aspartate aminotransferase (AST), albumin (ALB), globulin (GLB), and γ-glutamyl transpeptidase (γ-GGT). The blood samples were centrifuged at 4 °C and 4000 r/min for 10 min to separate the serum, which was then stored at −20 °C. Liver tissues were collected and processed as follows: (1) Approximately 3 g of liver tissue was frozen in RNase-free cryovials and stored at −80 °C for biochemical index detection; (2) 1–2 cm^3^ liver tissue samples were fixed in 4% neutral formalin for Hematoxylin-Eosin (HE) staining and Oil Red O staining observation; (3) Mung bean-sized liver tissue samples were quickly snap-frozen in liquid nitrogen and stored at −80 °C for molecular detection and lipidomic analysis. Pre-cooled normal saline was added to frozen liver tissues at a 1:1 weight-to-volume ratio, and after grinding and centrifugation at 3000 rpm for 20 min, the supernatant was collected and stored at −20 °C for biochemical testing.

### 2.5. Detection Indicators and Methods

#### 2.5.1. Liver Function Indicators

The total protein (TP), alanine aminotransferase (ALT), aspartate aminotransferase (AST), albumin (ALB), globulin (GLB), and γ-glutamyl transpeptidase (γ-GGT) were detected in the serum using an automatic biochemical analyzer Hitachi 7100 (Hitachi, Tokyo, Japan).

#### 2.5.2. Liver Oxidative Stress Indicators

The prepared liver tissue supernatant was thawed at room temperature to measure the contents of superoxide dismutase (SOD), total antioxidant capacity (T-AOC), malondialdehyde (MDA), and reactive oxygen species (ROS) in the liver using kits purchased from Nanjing Jiancheng Bioengineering Institute, Nanjing, China. All procedures were performed strictly according to the manufacturer’s instructions.

#### 2.5.3. Liver Inflammatory Indicators

Liver tissue supernatant was thawed at room temperature, and the interleukin-6 (IL-6), interleukin-8 (IL-8), interleukin-10 (IL-10), and tumor necrosis factor-α (TNF-α) levels were measured using kits purchased from Nanjing Jiancheng Bioengineering Institute, Nanjing, China. All operations were performed strictly according to accompanying instructions.

#### 2.5.4. Liver Histological Examination

Liver tissue blocks were fixed in 4% neutral formalin solution for 48 h, and dehydrated with graded ethanol, vitrified by dimethylbenzene, and embedded in paraffin. The paraffin blocks were sectioned into 4–6 μm thick slices and stained with Hematoxylin-Eosin (H&E) and Oil Red O, respectively. Histological changes and fat distribution in the liver were observed under an optical microscope.

#### 2.5.5. Liver Lipidomic Analysis

Approximately 30 mg of liver tissue stored at −80 °C was placed into a 2 mL centrifuge tube with 280 μL of methanol-water extraction solution (2:5, *V*/*V*) and 400 μL of methyl tert-butyl ether (MTBE). The mixture was ground at −10 °C and 50 Hz for 6 min, followed by ultrasonic extraction at 5 °C and 40 kHz for 30 min. Samples were allowed to stand at −20 °C for 30 min, whereafter the mixture was centrifuged at 4 °C and 13,000× *g* for 15 min. Then, 350 μL of the supernatant was collected and dried under a nitrogen flow. The residue was redissolved in 100 μL of isopropanol-acetonitrile solution (1:1, *V*/*V*), vortexed for 30 s, and ultrasonically extracted at 5 °C and 40 kHz for 5 min. After centrifugation at 4 °C and 13,000× *g* for 10 min, the supernatant was transferred to an injection vial with an inner tube for LC-MS analysis. An aliquot of 20 μL of supernatant from each sample per group was mixed to serve as a quality control (QC) sample. During analysis, a QC sample was injected after every 5–15 sample injections. Chromatographic separation was performed on an Accucore C30 column (100 mm × 2.1 mm i.d., 2.6 μm; Thermo Fisher Scientific, Waltham, MA, USA), and the gradient elution program is detailed in Table A1. Mass spectrometry was performed on a Q Exactive mass spectrometer (Thermo Fisher Scientific, Waltham, MA, USA) equipped with an electrospray ionization source, and data were collected under positive and negative ion modes, the parameters of which were detailed in Table A2. The collected raw data were imported into Lipidsearch (Thermo Fisher Scientific, Waltham, MA, USA), and after peak extraction, alignment, and identification, a data matrix containing peak retention times, peak areas, mass-to-charge ratios, and identification information was obtained for subsequent analysis.

#### 2.5.6. mRNA Detection of Heat Shock Proteins and Oxidative Stress-Related Factors

Total RNA was extracted from liver samples using the RNAiso Plus Kit (Cat. No. 9108, Takara Bio, Dalian, China) according to the manufacturer’s instructions. Reverse-transcription of RNA into cDNA was performed with PrimeScript™ RT reagent Kit with gDNA Eraser (Cat. No. RR047A, Takara Bio, Dalian, China) according to the manufacturer’s instructions. Real-time PCR was carried out using SYBR^®^ Premix Ex Taq™ II (Tli RNaseH Plus, Cat. No. RR820A, Takara Bio, Dalian, China) according to the manufacturer’s instructions. The primers for the target genes nuclear factor erythroid 2-related factor 2 (Nrf2), Heat Shock Protein 60 (HSP60), Heat Shock Protein 70 (HSP70), and Heat Shock Factor (HSF-1), and the internal reference gene Glyceraldehyde-3-phosphate dehydrogenase (GAPDH), were designed and synthesized by Sangon Biotech (Shanghai) Co., Ltd. (Shanghai, China) (Table 2). The relative expression levels of the target genes were calculated using the 2^(−ΔΔCt)^ method.

### 2.6. Statistical Analysis

The normality of data distribution was assessed using the Shapiro–Wilk test before one-way ANOVA, and homogeneity of variance was also examined. Growth performance, liver function indicators, oxidative stress indices, inflammatory factors, and gene expression data were analyzed by one-way ANOVA using IBM SPSS 26.0 (IBM Corp., Armonk, NY, USA), followed by Duncan’s multiple comparison test when significant differences were detected. For non-targeted lipidomic analysis, relative standard deviation (RSD) analysis was used to evaluate the reproducibility and stability of quality control (QC) samples. Principal component analysis (PCA) was performed to evaluate the overall distribution and clustering of samples, and orthogonal partial least-squares discriminant analysis (OPLS-DA) was used to further identify differences between groups. Differential metabolites were screened based on pairwise comparisons using an independent samples *t*-test, together with VIP > 1 and FC > 1. Graphs were plotted using GraphPad Prism 9.0 (GraphPad Software, San Diego, CA, USA). Significance was set at *p* < 0.05, while *p* < 0.01 indicated an extremely significant difference.

## 3. Results

### 3.1. Behavioral Observation and Physiological Changes

**Figure 1:**
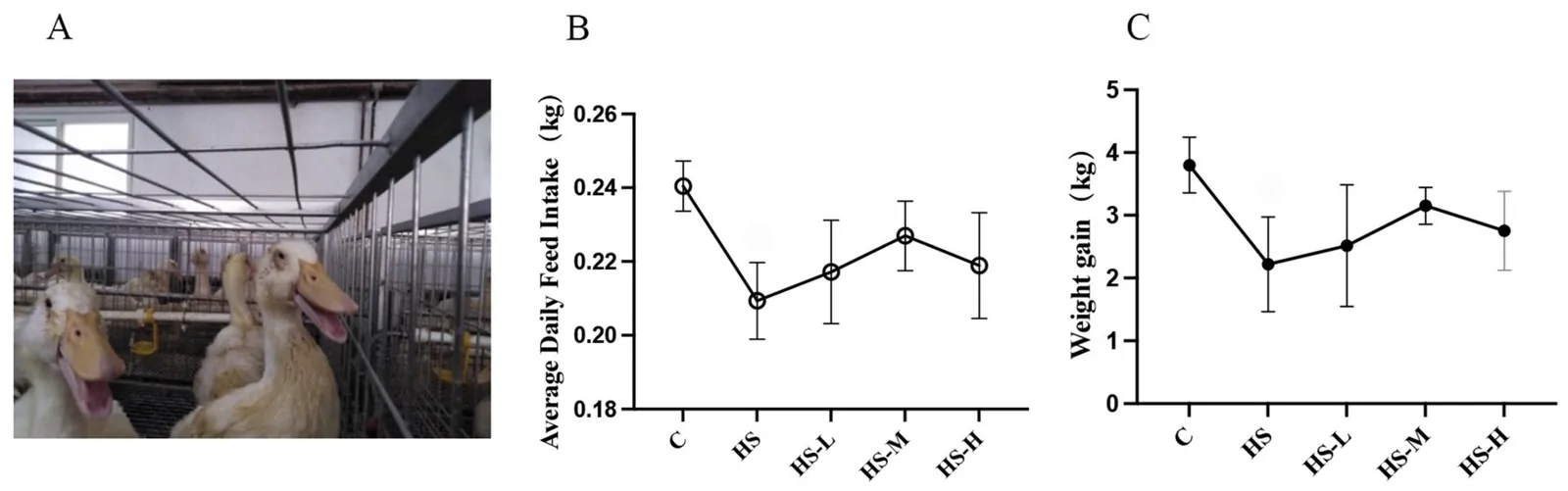
Effects of silybin on general behavioral and physiological changes in heat-stressed Peking ducks. (**A**): Heat-stressed Peking ducks; (**B**): average daily feed intake; (**C**): body weight gain. C: Control group; HS: heat-stress group; L + HS: 400 mg/kg silybin plus heat-stress group; M + HS: 800 mg/kg silybin plus heat-stress group; H + HS: 1600 mg/kg silybin plus heat-stress group.

As shown in Figure 1, Peking ducks exposed to heat stress showed panting, depression, and reduced activity. Compared with the C group, daily feed intake was significantly decreased in the HS group (*p* < 0.05). Silybin supplementation partially alleviated the heat-stress-induced decrease in daily feed intake, but the differences between the HS group and the silybin-supplemented groups were not statistically significant (*p* > 0.05). Similarly, body weight gain was significantly reduced in the HS group compared with the C group (*p* < 0.05). Although silybin supplementation partially alleviated the reduction in body weight gain, no significant differences were observed between the HS group and the silybin-supplemented groups (*p* > 0.05).

### 3.2. The Effect of Silybin on Liver Indicators of Peking Ducks Under Heat Stress

**Figure 2:**
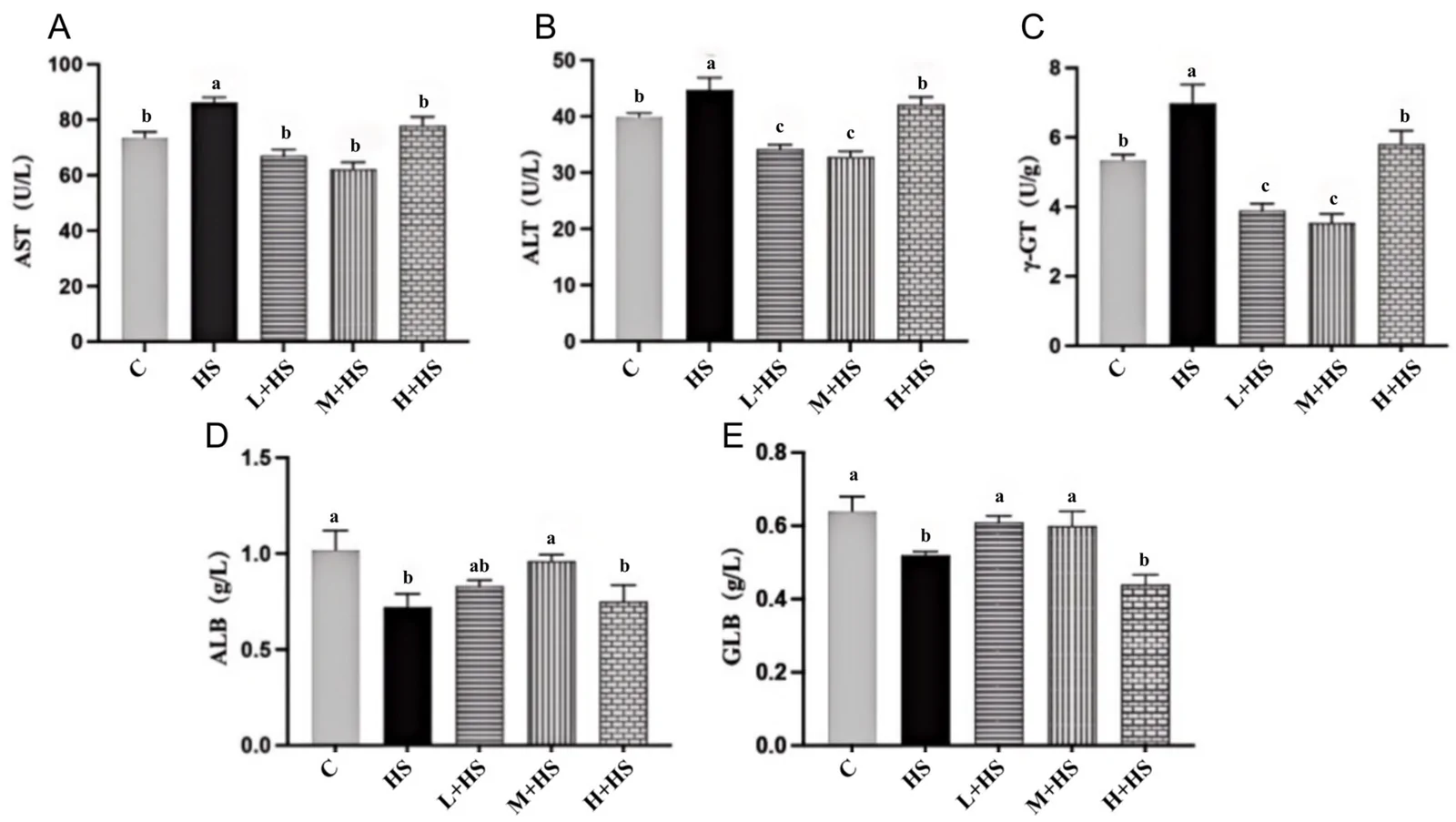
Effects of silybin on liver function indicators in heat-stressed Peking ducks. (**A**): Aspartate aminotransferase (AST); (**B**): alanine aminotransferase (ALT); (**C**): γ-glutamyl transpeptidase (γ-GGT); (**D**): albumin (ALB); (**E**): globulin (GLB). CON: Control group; HS: heat-stress group; L + HS: 400 mg/kg silybin plus heat-stress group; M + HS: 800 mg/kg silybin plus heat-stress group; H + HS: 1600 mg/kg silybin plus heat-stress group. Bars with different lowercase letters indicate significant differences among treatment groups (*p* < 0.05).

As shown in Figure 2, heat stress significantly affected liver function indicators in Peking ducks. Compared with the C group, AST, ALT, and γ-GGT levels were increased in the HS group, whereas ALB and GLB levels were decreased (*p* < 0.05), indicating that heat stress caused liver injury in Peking ducks. Compared with the HS group, the L + HS and M + HS groups showed lower AST, ALT, and γ-GGT levels (*p* < 0.05), indicating that 400 and 800 mg/kg silybin alleviated the heat-stress-induced increases in these liver injury markers. The H + HS group showed lower AST than the HS group, but its ALT and γ-GGT levels were higher than those in the L + HS and M + HS groups. For protein indicators, ALB was improved mainly in the M + HS group, while GLB was increased in the L + HS and M + HS groups compared with the HS group (*p* < 0.05). These results suggest that low- and medium-dose silybin alleviated heat-stress-induced liver damage more consistently than the high dose.

### 3.3. The Effect of Silybin on the Liver’s Antioxidant Capacity of Peking Ducks Under Heat Stress

**Figure 3:**
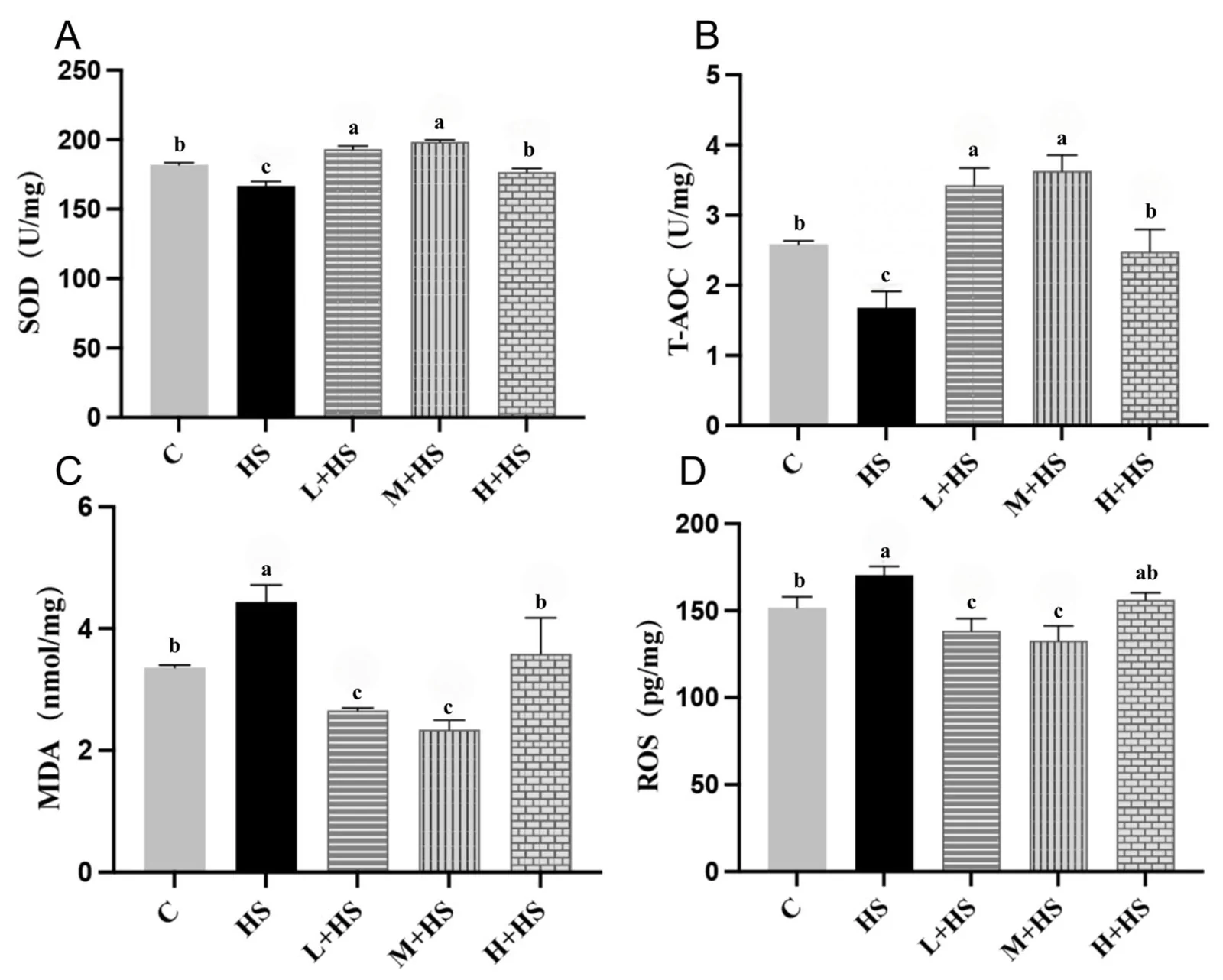
Effects of silybin on hepatic antioxidant indicators in heat-stressed Peking ducks. (**A**): Superoxide dismutase (SOD); (**B**): total antioxidant capacity (T-AOC); (**C**): malondialdehyde (MDA); (**D**): reactive oxygen species (ROS). C: Control group; HS: heat-stress group; L + HS: 400 mg/kg silybin plus heat-stress group; M + HS: 800 mg/kg silybin plus heat-stress group; H + HS: 1600 mg/kg silybin plus heat-stress group. Bars with different lowercase letters indicate significant differences among treatment groups (*p* < 0.05).

As shown in Figure 3, heat stress significantly affected hepatic antioxidant indicators in Peking ducks. Compared with the C group, SOD activity and T-AOC were decreased in the HS group, whereas MDA and ROS levels were increased (*p* < 0.05), indicating that heat stress induced oxidative stress in the liver. Compared with the HS group, the L + HS and M + HS groups showed increased SOD activity and T-AOC and decreased MDA and ROS levels (*p* < 0.05). The H + HS group also showed improvement in SOD activity, T-AOC, and MDA levels, but the response was less consistent than that observed in the L + HS and M + HS groups. These results indicate that silybin supplementation, especially at 400 and 800 mg/kg, alleviated heat-stress-induced oxidative damage in the liver of Peking ducks.

### 3.4. The Effect of Silybin on the Levels of Inflammatory Factors in the Liver of Peking Ducks Under Heat Stress

**Figure 4:**
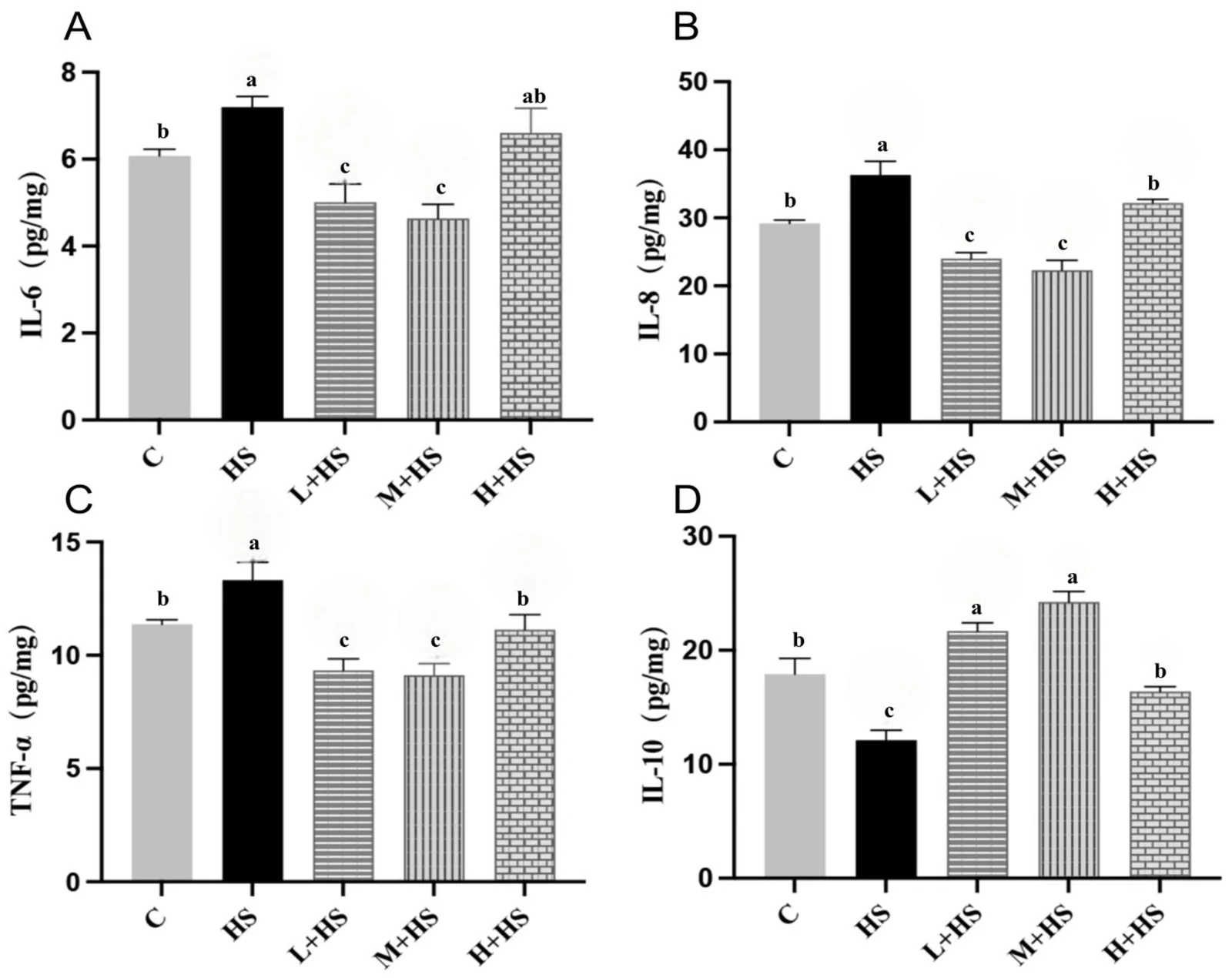
Effects of silybin on hepatic inflammatory factors in heat-stressed Peking ducks. (**A**): Interleukin-6 (IL-6); (**B**): interleukin-8 (IL-8); (**C**): tumor necrosis factor-α (TNF-α); (**D**): interleukin-10 (IL-10). C: Control group; HS: heat-stress group; L + HS: 400 mg/kg silybin plus heat-stress group; M + HS: 800 mg/kg silybin plus heat-stress group; H + HS: 1600 mg/kg silybin plus heat-stress group. Bars with different lowercase letters indicate significant differences among treatment groups (*p* < 0.05).

As shown in Figure 4, heat stress significantly affected hepatic inflammatory factors in Peking ducks. Compared with the C group, IL-6, IL-8, and TNF-α levels were increased in the HS group, whereas IL-10 level was decreased (*p* < 0.05), indicating that heat stress induced hepatic inflammatory responses. Compared with the HS group, the L + HS and M + HS groups showed lower IL-6, IL-8, and TNF-α levels and higher IL-10 levels (*p* < 0.05). The H + HS group also showed lower IL-8 and TNF-α levels and higher IL-10 levels than the HS group, whereas the reduction in IL-6 was less evident. These results suggest that silybin supplementation moderated heat-stress-induced inflammatory responses in the liver of Peking ducks, with more consistent effects in the low- and medium-dose groups.

### 3.5. The Effect of Silybin on the Liver Tissue Structure of Peking Ducks Under Heat Stress

**Figure 5:**
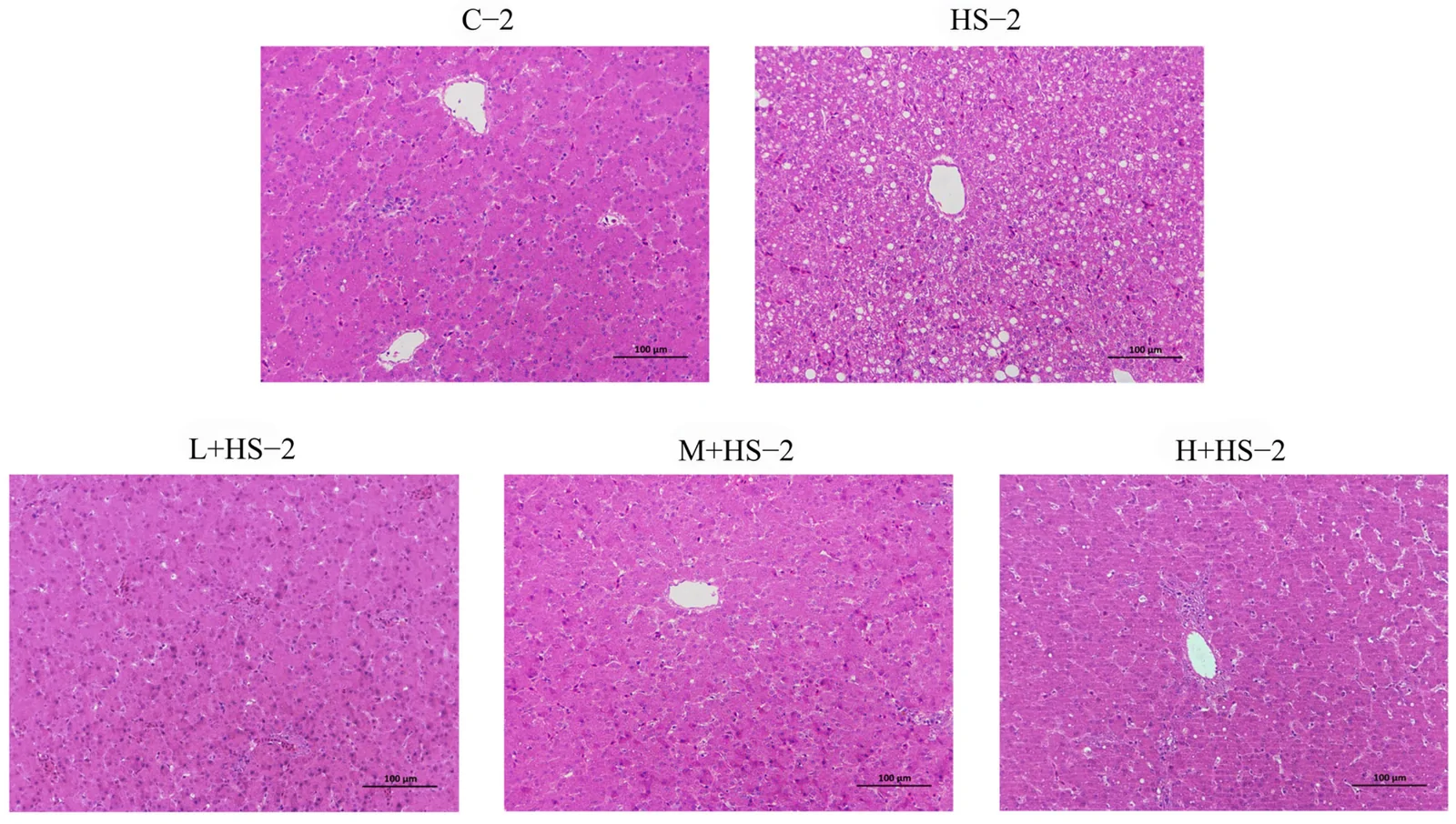
Effects of silybin on liver tissue structure in heat-stressed Peking ducks. C: Control group; HS: heat-stress group; L + HS: 400 mg/kg silybin plus heat-stress group; M + HS: 800 mg/kg silybin plus heat-stress group; H + HS: 1600 mg/kg silybin plus heat-stress group. Images labeled −2 are shown at 200× magnification, and images labeled −1 are shown at 100× magnification.

**Figure 6:**
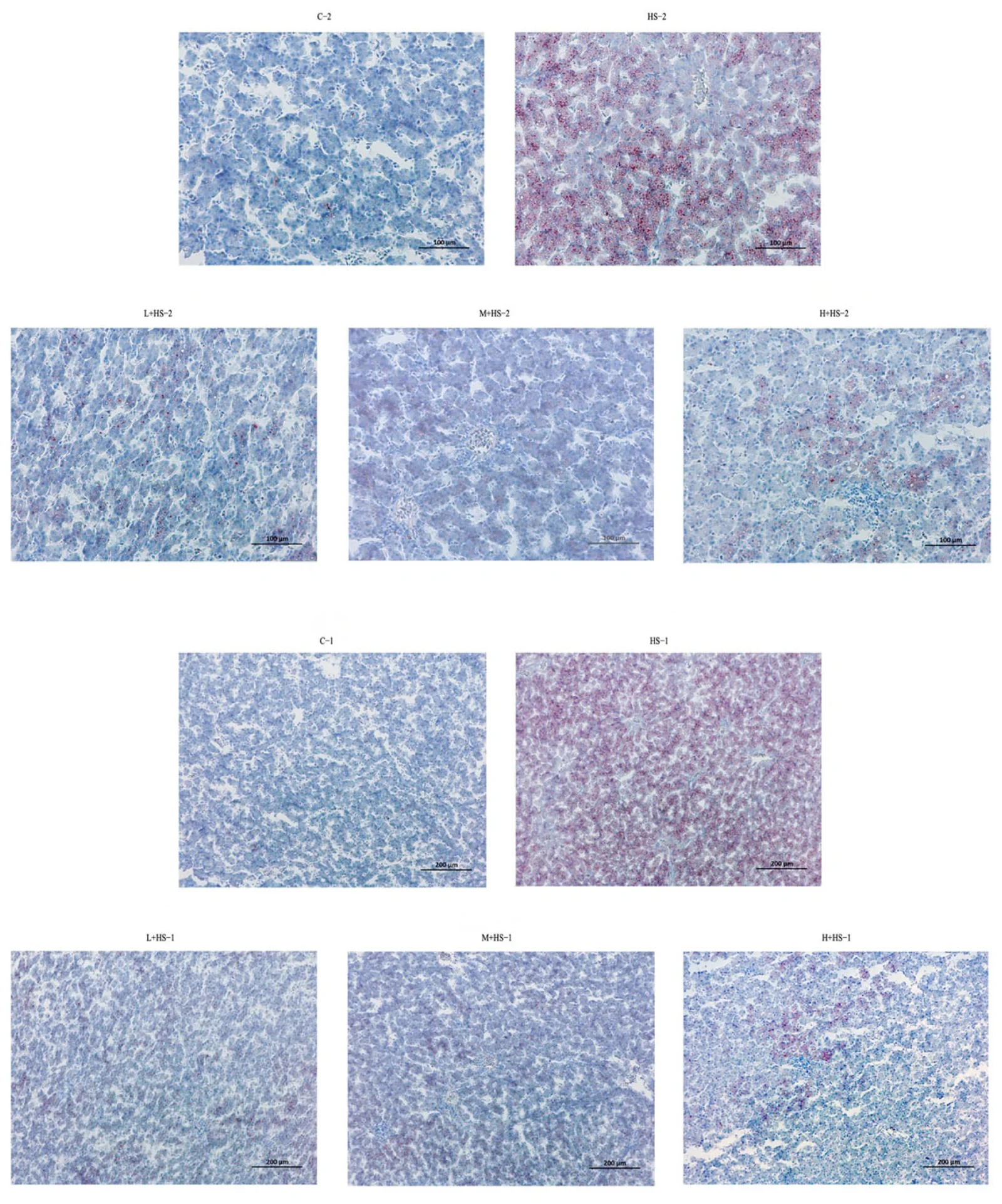
Effects of silybin on liver steatosis in heat-stressed Peking ducks. C: Control group; HS: heat-stress group; L + HS: 400 mg/kg silybin plus heat-stress group; M + HS: 800 mg/kg silybin plus heat-stress group; H + HS: 1600 mg/kg silybin plus heat-stress group. Images labeled −2 are shown at 100× magnification, and images labeled −1 are shown at 200× magnification.

As shown in Figure 5, the liver tissue structure of the C group was normal, with orderly arranged liver cells. After heat stress, the basic liver structure of the Peking ducks collapsed, with numerous vacuolar-like structures present in the cytoplasm visible under the microscope, along with local congestion and inflammatory infiltration. In the L + HS, M + HS, and H + HS groups, the vacuolar-like structures in the liver decreased, and the cell arrangement became more orderly. The liver cell cords became clearer, and the infiltration of inflammatory cells improved. As shown in Figure 6, the livers of the C group also showed normal histology with no signs of steatosis, while those of the HS group revealed severe fatty degeneration with a significant accumulation of fat. Results confirmed that the addition of silybin could alleviate heat-stress-induced fatty degeneration in the liver of Peking ducks.

### 3.6. The Effects of Silybin on the mRNA Expression of HSPs and Nuclear Transcription Factor-Related Factors in Heat-Stressed Peking Ducks

**Figure 7:**
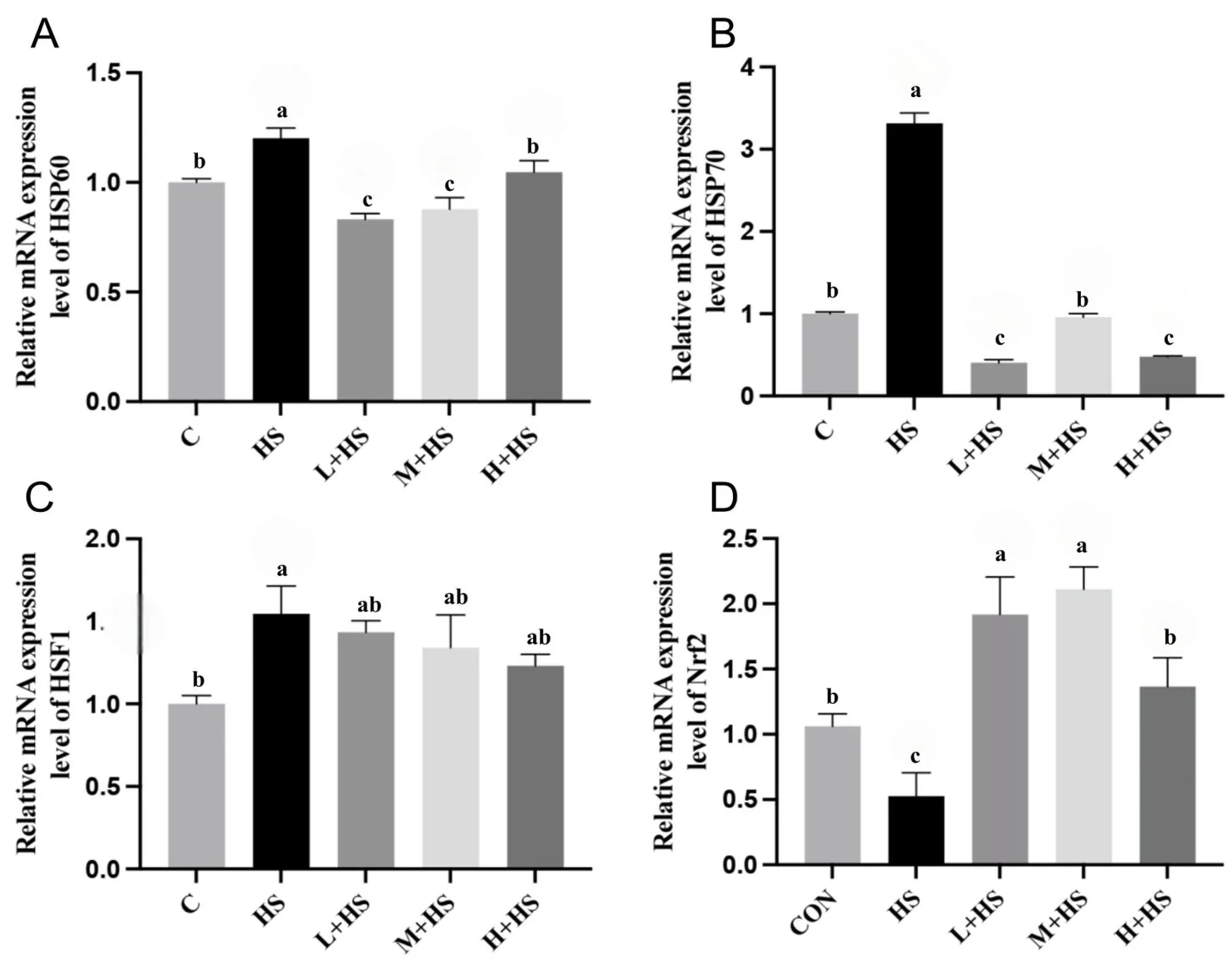
Effects of silybin on the hepatic mRNA expression of heat shock proteins and oxidative stress-related genes in heat-stressed Peking ducks. (**A**): Heat shock protein 60 (HSP60); (**B**): heat shock protein 70 (HSP70); (**C**): heat shock factor 1 (HSF1); (**D**): nuclear factor erythroid 2-related factor 2 (Nrf2). C/CON: control group; HS: heat-stress group; L + HS: 400 mg/kg silybin plus heat-stress group; M + HS: 800 mg/kg silybin plus heat-stress group; H + HS: 1600 mg/kg silybin plus heat-stress group. Bars with different lowercase letters indicate significant differences among treatment groups (*p* < 0.05).

As shown in Figure 7, heat stress significantly affected the hepatic mRNA expression of heat shock proteins and oxidative stress-related genes in Peking ducks. Compared with the C group, HSP60, HSP70, and HSF1 mRNA expression levels were increased in the HS group, whereas Nrf2 mRNA expression was decreased (*p* < 0.05). Compared with the HS group, silybin supplementation decreased HSP60 and HSP70 mRNA expression and increased Nrf2 mRNA expression (*p* < 0.05). The expression of HSF1 tended to decrease after silybin supplementation, but the differences among the HS and silybin-supplemented groups were not significant. These results suggest that silybin regulated heat-stress-related gene expression, especially HSP60, HSP70, and Nrf2, in the livers of heat-stressed Peking ducks.

### 3.7. Total Ion Chromatogram of QC Samples and Sample Quality Control

**Figure A1:**
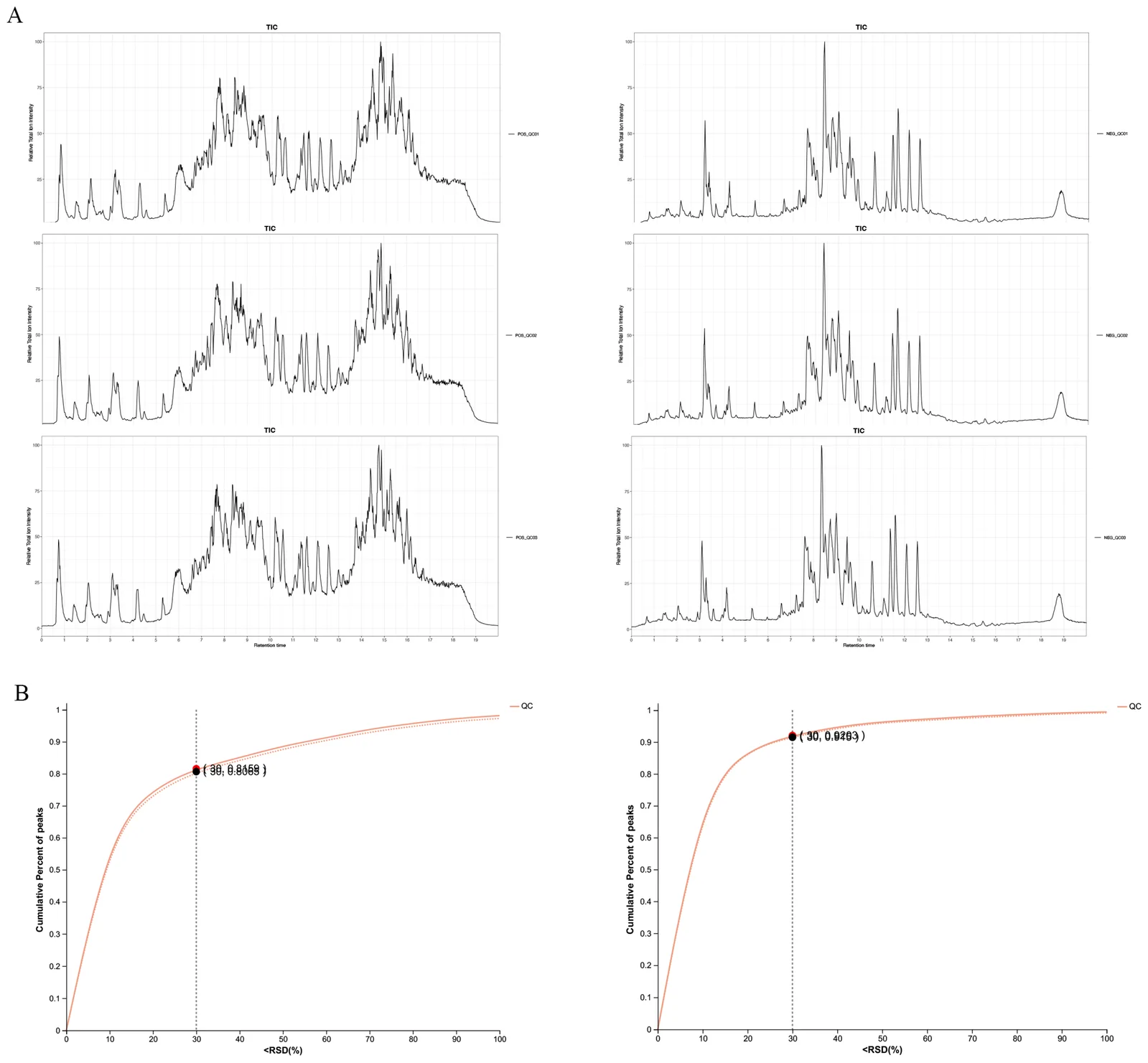
Total ion superposition chromatogram and relative standard deviation chart of QC samples. Left shows in positive ion mode, and right shows in negative ion mode. (**A**): Total ion stacking chromatogram; (**B**): Relative standard deviation chart.

Based on the results of the macroscopic indicator tests, liver tissues from the C, HS, and L + HS groups were selected for subsequent metabolomics analysis. As shown in Figure A1, the total ion chromatograms of the QC samples showed high overlap in both positive and negative ion modes, indicating good reproducibility of metabolites and instrument stability. Relative standard deviation (RSD) analysis showed that the cumulative proportion of ion peaks with RSD < 30% was >70%. This result meets the criteria for data quality control, indicating good repeatability and stability of the analysis, and supporting the use of the data in further investigation.

### 3.8. PCA and OPLS-DA Analysis

**Figure 8:**
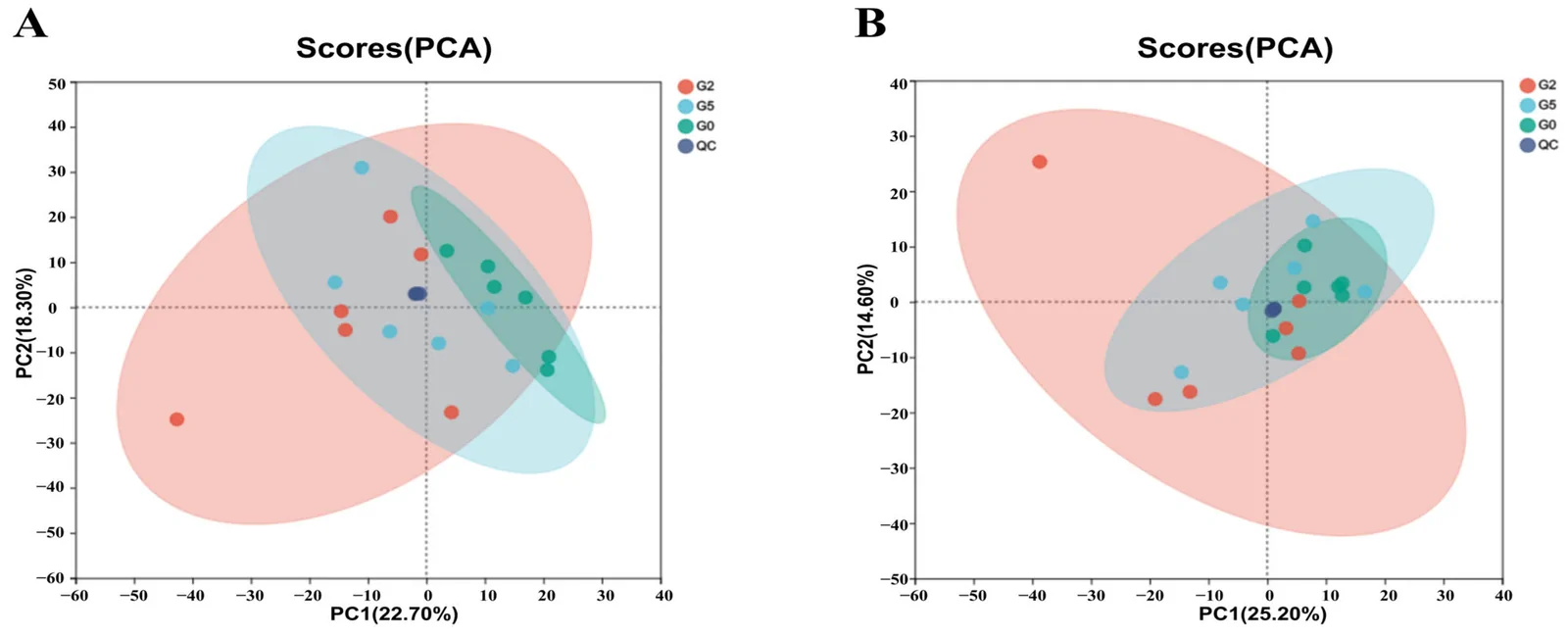
PCA of liver lipidomic samples. (**A**): PCA in positive-ion mode; (**B**): PCA in negative-ion mode. C: Control group; HS: heat-stress group; L + HS: 400 mg/kg silybin plus heat-stress group; QC: quality control sample; PCA: principal component analysis.

**Figure 9:**
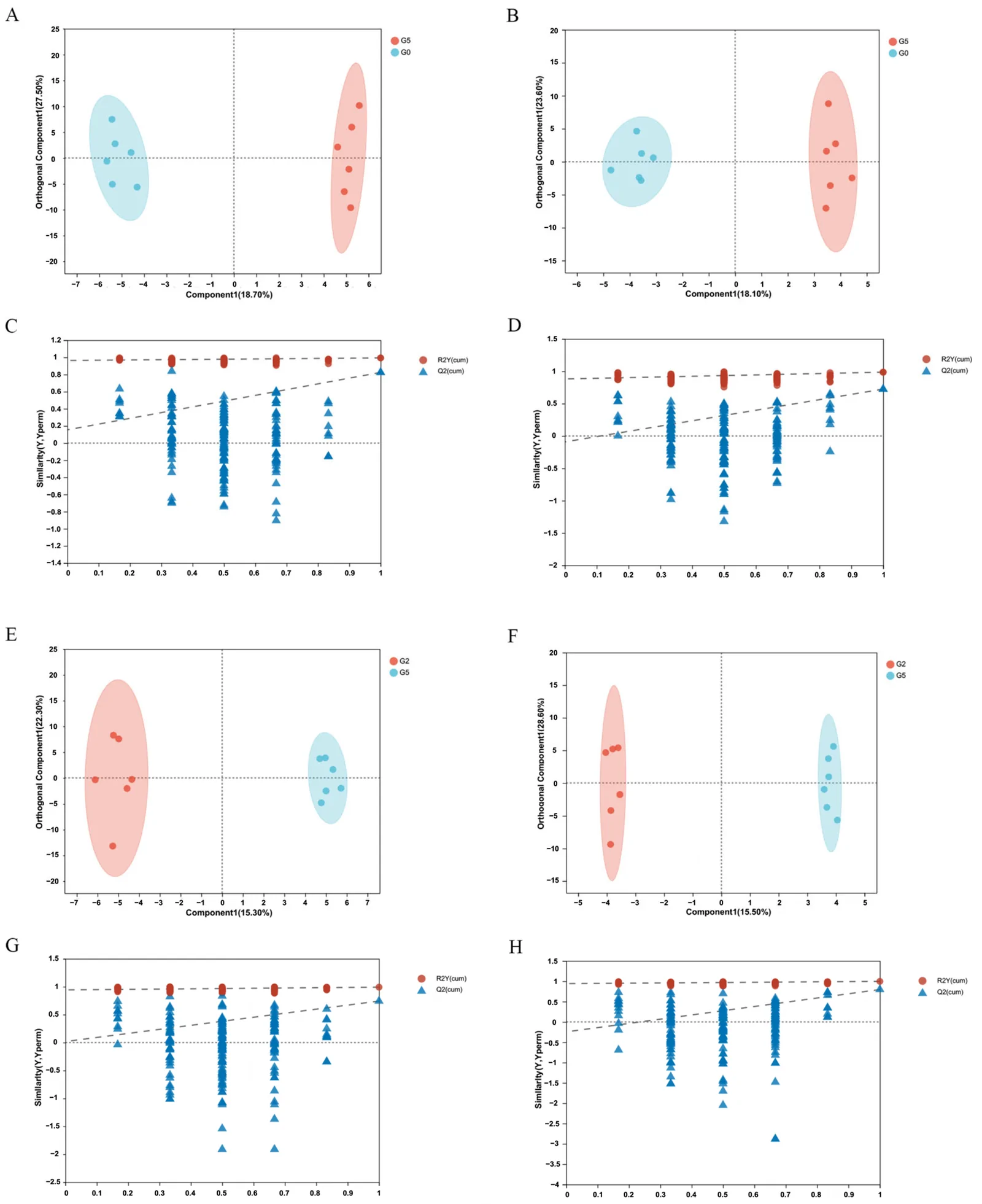
OPLS-DA analysis of liver lipidomic samples. (**A**,**E**) are OPLS-DA score plots of C vs. HS and HS vs. L + HS in positive-ion mode, respectively; (**B**,**F**) are OPLS-DA score plots of C vs. HS and HS vs. L + HS in negative-ion mode, respectively; (**C**,**G**) are permutation tests of C vs. HS and HS vs. L + HS in positive-ion mode, respectively; (**D**,**H**) are permutation tests of C vs. HS and HS vs. L + HS in negative-ion mode, respectively. C: Control group; HS: heat-stress group; L + HS: 400 mg/kg silybin plus heat-stress group; OPLS-DA: orthogonal partial least-squares discriminant analysis.

As shown in Figure 8, the PCA revealed that the QC samples clustered tightly in both the positive-ion (Figure 8A) and negative-ion (Figure 8B) modes, demonstrating good instrumental stability and data reliability. However, there was some overlap between the samples in each group, leading to complete separation between groups, and further analysis was conducted using OPLS-DA. The evaluation parameters of the OPLS-DA model are shown in Table A3, where R2Y represents the model’s explanatory ability, and Q2 represents its predictive ability. The intercept of the Q2 regression line with the Y-axis was less than 0.05, indicating a robust and reliable model without overfitting, and could thus be used for subsequent analysis. Since there was no crossover or repetition among different treatment groups, and the degree of separation was significant, classification is clear (Figure 9). Both the intercepts of the Q2 regression line with the Y-axis were less than 0.05, indicating that the permutation test was valid and the model was not overfitted.

### 3.9. Screening of Differential Metabolites

**Figure 10:**
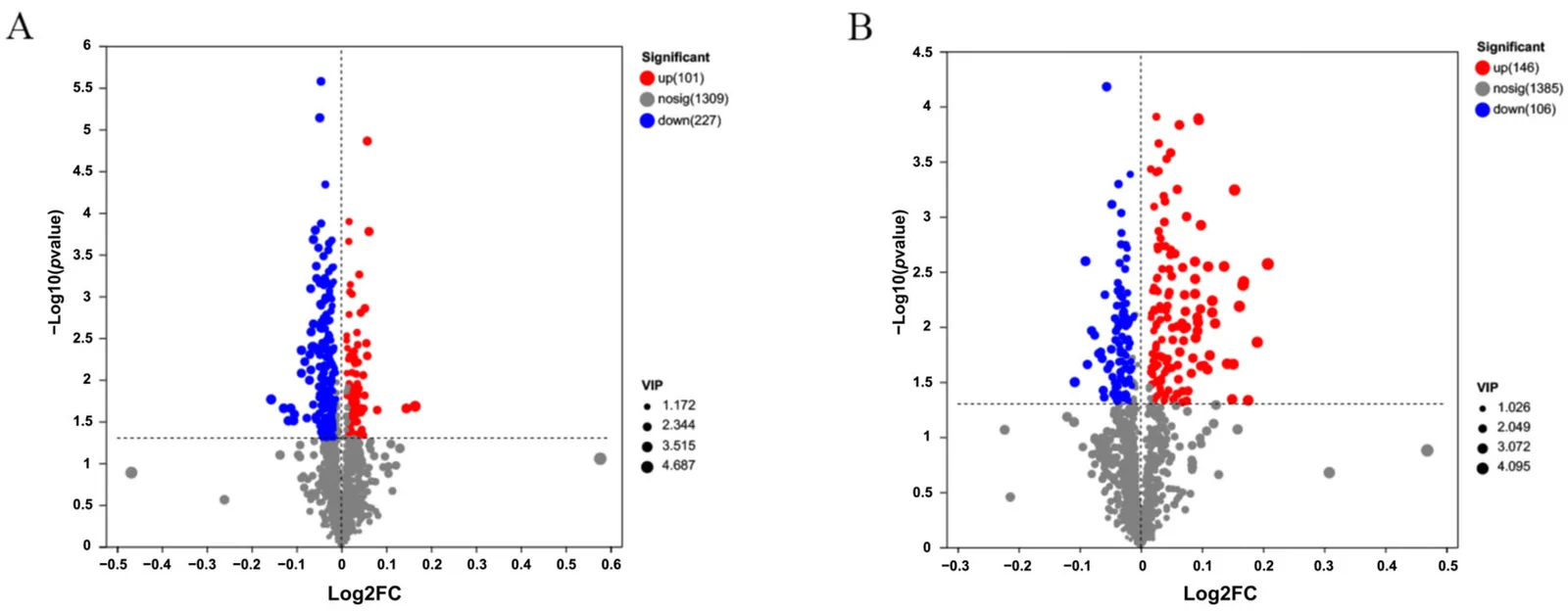
Volcano plot of differential metabolites. (**A**): Volcano plot of differential metabolites between C and HS groups; (**B**): volcano plot of differential metabolites between HS and L + HS groups. C: Control group; HS: heat-stress group; L + HS: 400 mg/kg silybin plus heat-stress group.

Based on the OPLS-DA model, differential metabolites were screened by *p*-value, VIP value, and fold change in expression. *p* < 0.05, VIP > 1, and FC > 1 were set as the criteria, and differential metabolites were screened from different treatment groups under different modes. As shown in Table 3 and Figure 10, a total of 328 differential metabolites were screened between the C and HS groups, including 199 metabolites in positive ion mode and 129 metabolites in negative ion mode. Among them, 101 were up-regulated with 53 in positive ion mode and 48 in negative ion mode, while 227 were down-regulated with 146 in positive ion mode and 81 in negative ion mode. A total of 252 differential metabolites were identified between the HS and L + HS groups, including 145 metabolites in positive ion mode and 107 metabolites in negative ion mode. Among them, 146 were up-regulated, with 90 in positive ion mode and 56 in negative ion mode, while 106 were down-regulated, with 55 in positive ion mode and 51 in negative ion mode.

### 3.10. Clustering Analysis of Differential Metabolites

**Figure 11:**
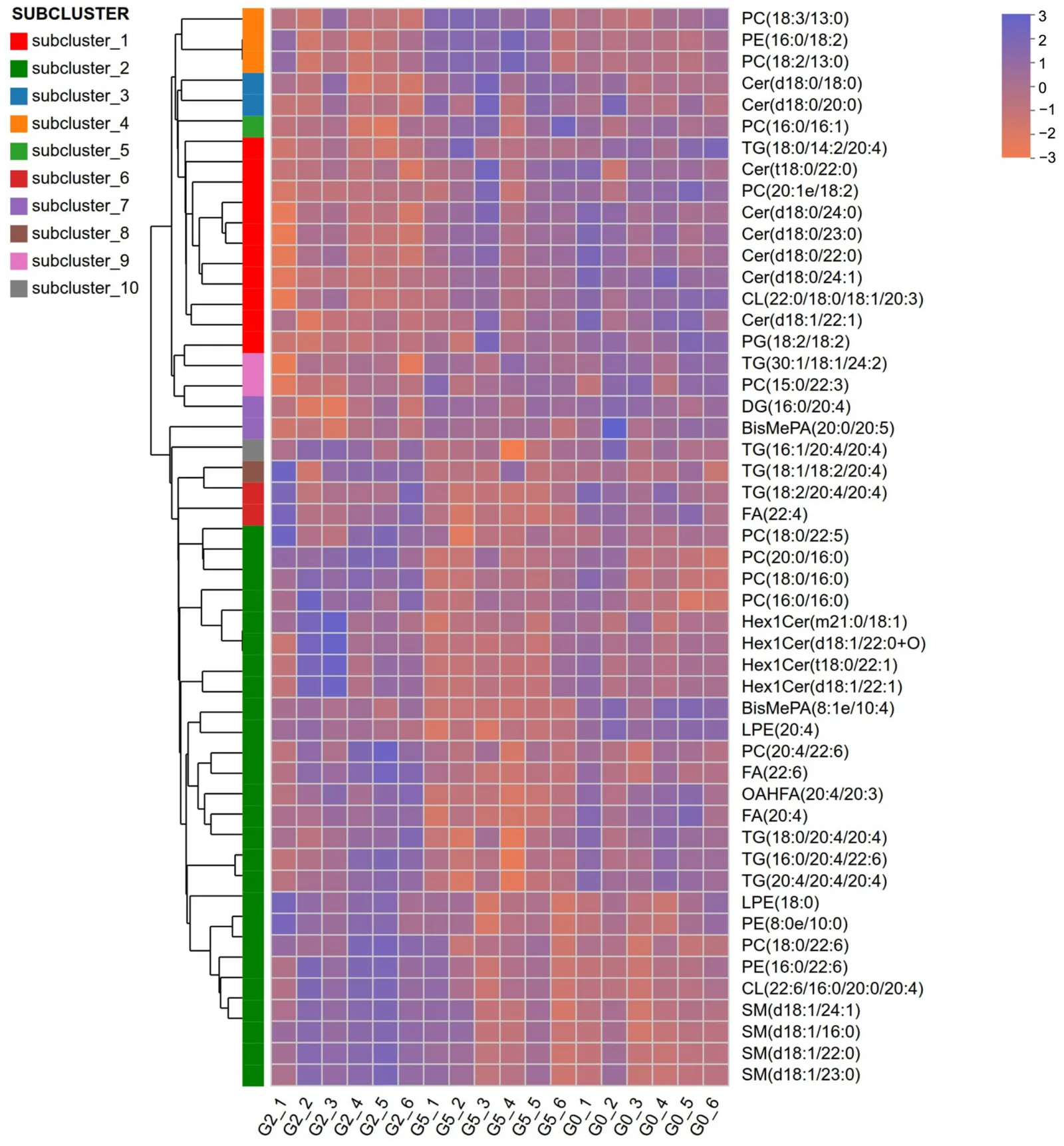
Heatmap for clustering analysis of differential metabolites between groups. A: Heatmap of C vs. HS group clustering analysis; B: heatmap of HS vs. L + HS group clustering analysis. C: Control group; HS: heat-stress group; L + HS: 400 mg/kg silybin plus heat-stress group.

**Figure 12:**
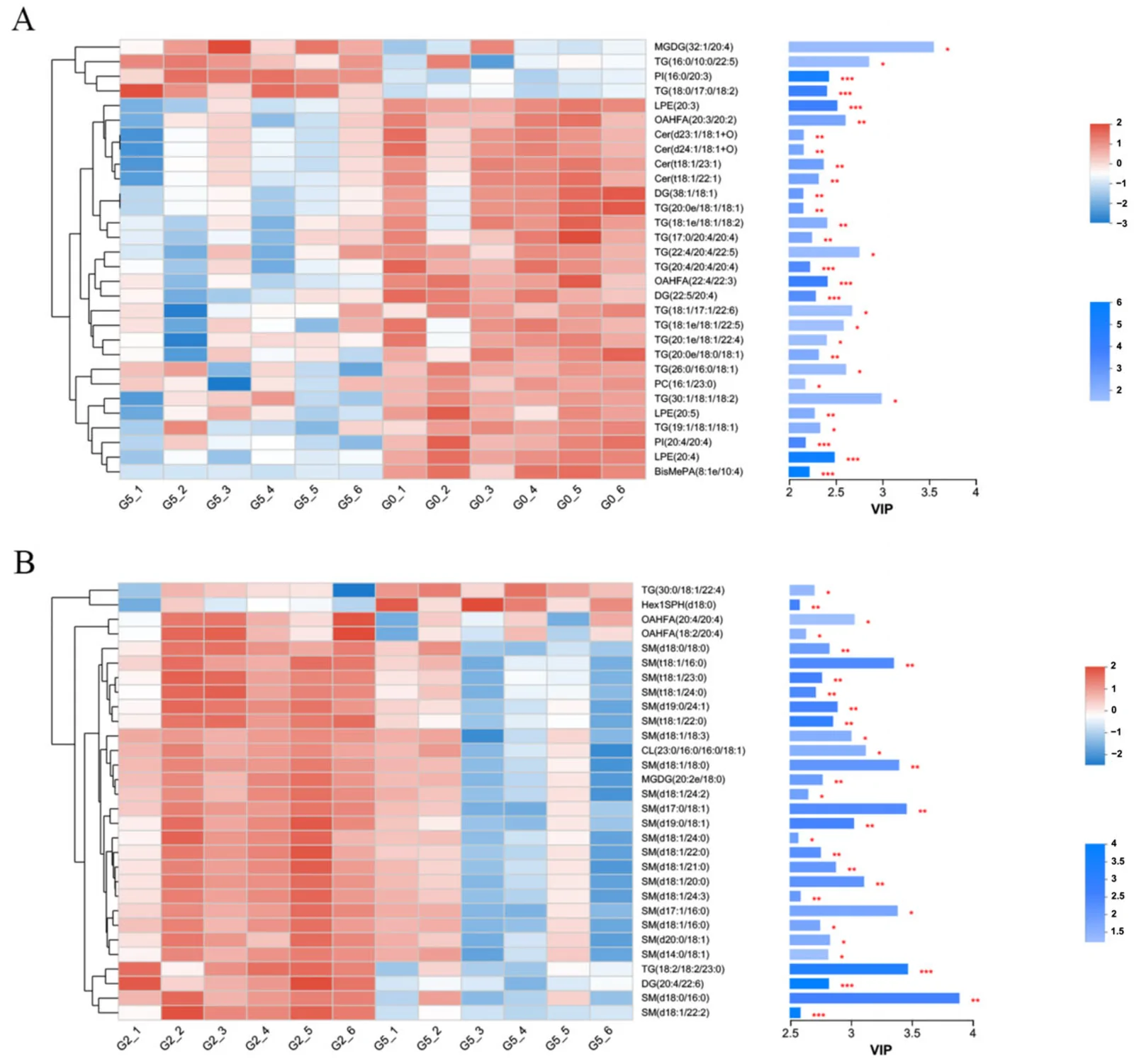
Top 30 significantly different metabolites based on VIP values in liver tissue samples. (**A**): Top 30 significantly different metabolites based on VIP values between C and HS groups; (**B**): top 30 significantly different metabolites based on VIP values between HS and L + HS groups. C: Control group; HS: heat-stress group; L + HS: 400 mg/kg silybin plus heat-stress group; VIP: variable importance in projection. * represents *p* < 0.05, ** represents *p* < 0.01, and *** represents *p* < 0.001.

The expression of metabolites across samples within the same group remained relatively consistent but differed from that in other groups (Figure 11). In the clustering diagram, closer metabolite branches show more similar expression levels. The top 30 differential metabolites were identified based on their VIP values (Table A4). As shown in Figure 12A, when metabolites were sorted by VIP value from high to low, the VIP values of all the top 30 differential metabolites between the C and HS groups were greater than 2, with significant differences. Compared with group C, 26 differential metabolites in the HS group were significantly down-regulated, while four were significantly up-regulated. As shown in Figure 12B, 28 differential metabolites in L + HS were significantly up-regulated, and two were significantly down-regulated. Furthermore, the top 30 significantly different metabolites between groups C and HS could be classified into four major categories according to the number of differential metabolites in each category in descending order; these were: Glycerolipids (GLs), Glycerophospholipids (GPs), Sphingolipids (SPs), and Fatty Acyls (FAs). The top 30 metabolites that differed significantly between groups HS and L + HS were also classified into four major categories according to the number of differential metabolites in each category in descending order. These were: Sphingolipids (SPs), Glycerolipids (GLs), Fatty Acyls (FAs), and Glycerophospholipids (GPs).

### 3.11. KEGG Pathway Enrichment Analysis of Differential Metabolites

**Figure 13:**
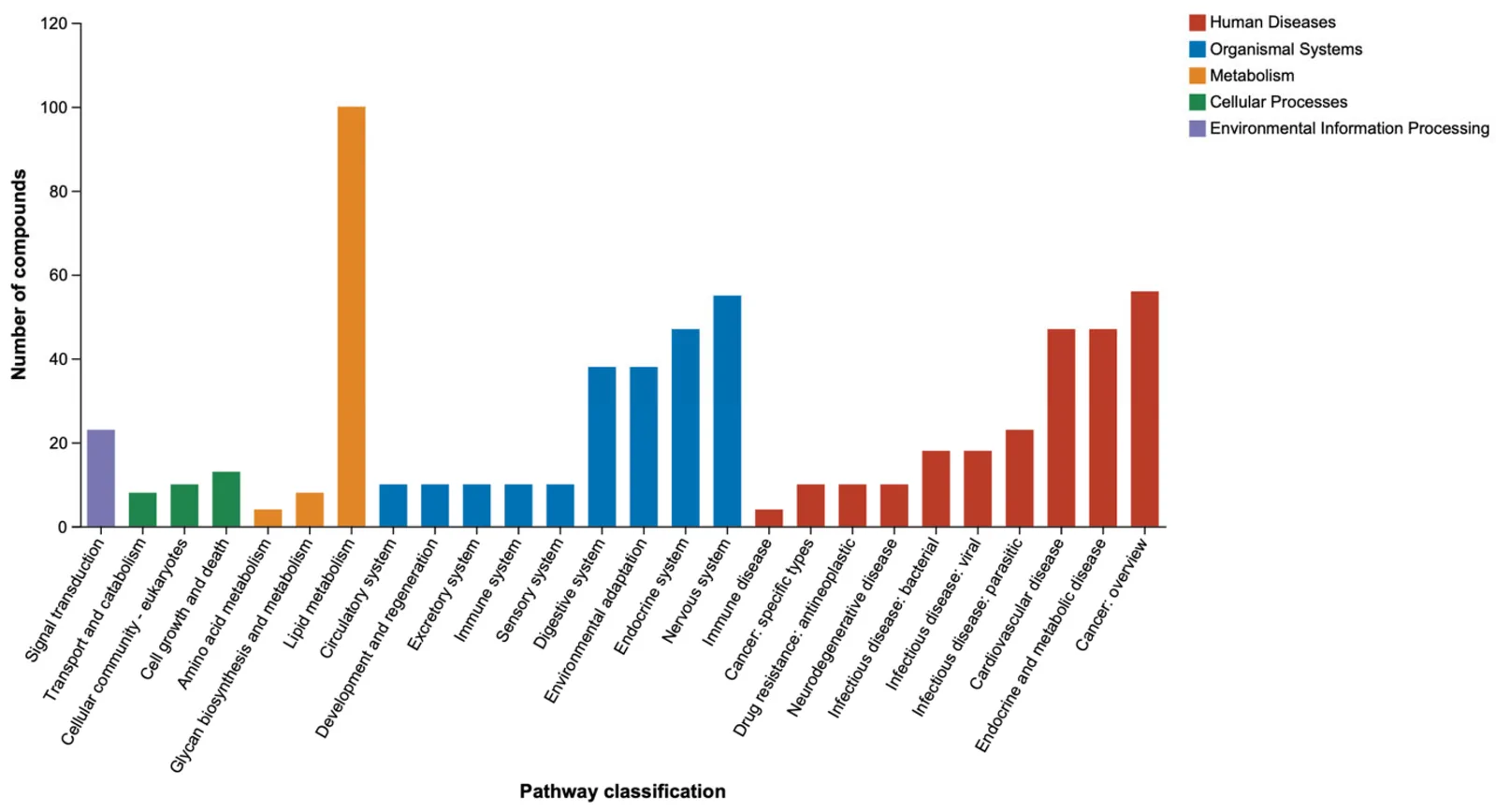
Metabolic pathway analysis of liver samples.

As shown in Figure 13, the KEGG pathway enrichment analysis results for all metabolites identified from liver tissue revealed pathways enriched in five categories: Human Diseases, Organismal Systems, Metabolism, Cellular Processes, and Environmental Information Processing. Within the category of metabolism, lipid metabolism accounted for the vast majority, while Amino acid metabolism and Glycan biosynthesis and metabolism accounted for only minor fractions. Within the category of Organismal Systems, Nervous System, Endocrine System, Environmental Adaptation, and Digestive System accounted for the most.

**Figure 14:**
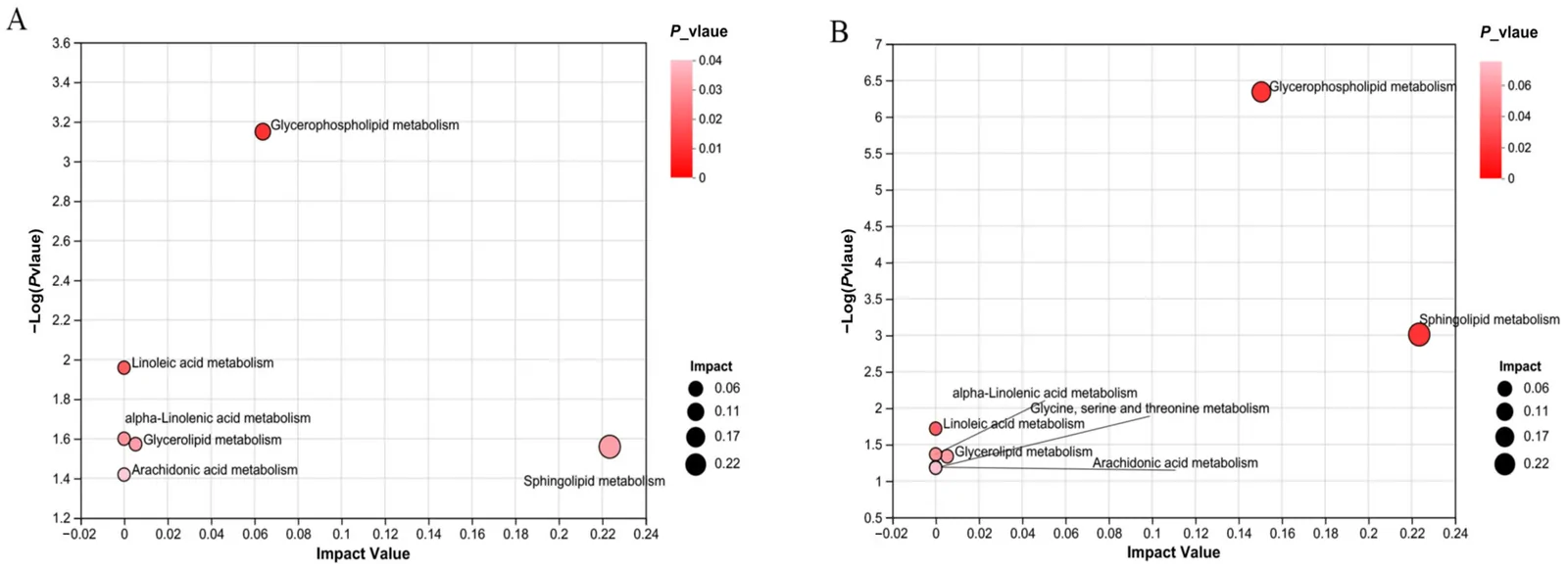
Bubble diagram of topological analysis of metabolic pathways. (**A**): Results of topological analysis of the C and HS groups; (**B**): results of topological analysis of the HS and L + HS groups. C: Control group; HS: heat-stress group; L + HS: 400 mg/kg silybin plus heat-stress group.

Given the complexity and diversity of lipid metabolism, the MetaboAnalyst 6.0 software (https://www.metaboanalyst.ca) was used to perform KEGG topological analysis of significantly different metabolites (Figure 14) to screen for lipid metabolism pathways that were most influential in the experiment. The results showed that all the differential metabolites in both comparison results were involved in six major lipid metabolism pathways (Table A6), where the total number of metabolites in each pathway was 33, 52, 32, 13, 30, and 46, respectively. Among these lipid metabolism pathways, the sphingolipid metabolism pathway was the most important, followed by the glycerophospholipid metabolic pathway and the glycerol ester metabolic pathway. Moreover, enrichment of the glycerophospholipid metabolic pathway was most significant. The above results indicated that the sphingolipid and glycerophospholipid metabolism pathways were the two most essential pathways through which heat stress affected liver lipid metabolism in Peking ducks. Importantly, they were also the most important pathways through which silybin regulated the liver lipid metabolism of the Peking ducks affected by heat stress.

## 4. Discussion

Inflammation is a normal defense mechanism of the body and plays a critical role in maintaining health, and under normal conditions, the secretion of pro-inflammatory and anti-inflammatory factors maintains a dynamic balance. The liver is one of the most commonly damaged organs due to heat stress [17]. Studies have found that heat stress-induced liver injury is often accompanied by activated inflammatory responses, which further induce the secretion of IL-1β, TNF-α, and IL-6 through direct stimulation or signaling cascades of NF-κB and TLR4, thereby exacerbating liver injury [8,18]. Guo et al. [19] found that heat stress led to increased body temperature, reduced nutrient absorption and utilization, and decreased growth performance in yellow-feather broilers, which was consistent with previous research results [7]. In this experiment, Peking ducks subjected to heat stress panted, spread their wings frequently, and showed decreased body weight and feed intake. Furthermore, compared with the control, heat stress significantly increased pro-inflammatory factors and decreased anti-inflammatory factors in the livers of Peking ducks. Notably, this was significantly reversed after silybin intervention, and the liver’s inflammatory response was markedly alleviated. The liver plays a crucial role in maintaining normal metabolic activities, and its healthy tissue structure is of great significance in maintaining its metabolic function. However, the liver is highly sensitive to external stimuli, which may disrupt the metabolic balance of the liver and even the entire body [20]. Ding et al. found that heat stress led to disordered hepatocyte arrangement and significantly enhanced inflammatory infiltration and local necrosis in broilers [21]. Similarly, current results revealed that the livers of the HS group showed numerous vacuolar-like structures, accompanied by local congestion and inflammatory cell infiltration, and a damaged liver tissue structure. Supplementation with silybin could significantly improve heat-stress-induced pathological liver damage under the same conditions and restore the basic liver tissue structure. Additionally, 400 and 800 mg/kg silybin supplementation reduced the activities of ALT, AST, and γ-GGT, while improvements in ALB and GLB were mainly observed in the 800 mg/kg group, and GLB was also increased in the 400 mg/kg group, thereby indicating a partial alleviation of heat-stress-induced liver damage in Peking ducks. Notably, the protective effects of silybin were not linearly dose-dependent. Compared with the low- and medium-dose groups, the H + HS group showed less favorable responses in several liver injury indicators, including AST, ALT, and γ-GGT. This may suggest a non-linear or biphasic dose–response pattern. Under heat-stress conditions, excessive silybin supplementation may not provide additional hepatic protection and may even increase hepatic metabolic burden. However, because the present study was not designed to evaluate silybin toxicity or determine the precise optimal dose, this explanation requires further verification in future studies.

Under high environmental temperatures in summer, poultry cannot effectively dissipate heat with poor air circulation. When tissues and organs are exposed to prolonged hyperthermia, the body’s oxygen demand increases, inducing the production of a large amount of ROS [22,23,24], which triggers oxidative damage to cellular macromolecules. When the damage exceeds the scavenging capacity of the body’s antioxidant enzymes, oxidative stress occurs, leading to liver injury [25,26]. Studies have shown a strong correlation between oxidative stress and liver injury [27]. Here, SOD is a key component of the antioxidant enzyme system, playing a crucial role in maintaining the balance between oxidation and antioxidation [21,28]. Here, T-AOC reflects the total antioxidant capacity of the body, including antioxidant enzymes [29]. Similarly, MDA is the final product of the peroxidation reaction triggered by a free radical attack on unsaturated fatty acids and can reflect the degree of oxidative damage caused by free radicals in the body [28]. In the livers of Peking ducks, heat stress resulted in a significant decrease in SOD and T-AOC and a significant increase in MDA and ROS levels, inducing a severe oxidative stress response, a finding consistent with results reported in other studies [21,30,31]. Silybin could significantly increase the content of SOD and T-AOC in the livers of heat-stressed Peking ducks, reducing the levels of MDA and ROS, and thus successfully alleviating oxidative stress caused by heat stress in the liver.

Heat stress proteins (HSPs) are widely distributed in organisms and are usually expressed at low levels, although their expression increases when the organism is under stress. These HSPs play an important role in resisting stress-induced damage and maintaining homeostasis, and their expression levels can reflect the body’s ability to resist stress-induced damage [32,33]. The expression of HSPs can enhance cellular thermotolerance and reduce the damage caused by heat stimulation [34]. Studies have reported that bamboo leaf flavonoids could reduce the mRNA expression of HSF1, HSP70, and HSP90 in BMECs, enhance cellular thermotolerance, and protect the normal function of cells under heat stress [35]. Research has found that heat stress led to a significant increase in hepatic HSP levels in mice, which was alleviated after a single dose of a traditional Chinese medicine [36]. In this study, heat stress induced an increase in the expression of HSP60, HSP70, and HSF1, and the expression of heat shock proteins decreased after silybin intervention, indicating that silybin maintained HSP homeostasis in the liver and protected the liver under heat stress conditions. Although the treatment and experimental animal were different, this result was consistent with previous studies showing that heat stress can induce HSP-related responses and that bioactive substances may regulate HSP expression under stress conditions [37].

Also, Nrf2 is closely related to the body’s oxidative stress and is a major regulatory factor that promotes cell survival by regulating the body’s oxidative and antioxidant states. Upon activation, Nrf2 reduces the body’s stress response by inducing the transcription of various antioxidant-related genes downstream, including HO-1 and NQO-1 [38]. Many bioactive substances alleviate liver injury by activating Nrf2, manifested as a decrease in serum levels of ALT, LDH, IL-1β, and ROS [21,39,40]. Studies have found that heat stress can significantly reduce the mRNA expression of Nrf2 and increase the mRNA expression of HSP70 in the liver of broilers [21], while resveratrol can reverse this. This is similar to findings of other studies [41,42]. In this study, the mRNA expression level of Nrf2 in the liver of Peking ducks in the HS group decreased significantly, while the mRNA expression of Nrf2 increased after silybin intervention, suggesting that silybin can alleviate liver damage caused by heat stress by activating the expression of Nrf2.

In addition to inflammatory and histopathological injury, hepatic lipid metabolism was also affected by heat stress. The primary site of fat synthesis in poultry is the liver, which plays a pivotal role in lipid metabolism. The liver is particularly vulnerable to damage under heat stress owing to its high sensitivity to external stimuli. Current results showed that heat stress disrupted the normal structure and fat metabolism function of the liver of Peking ducks, resulting in a large number of vacuolar-like structures, pathological changes such as congestion and inflammatory infiltration, extensive lipid droplet accumulation, and severe fat degeneration, consistent with the previous research results [12,21]. Heat stress was shown to cause a significant increase in the level of fat synthase in the liver and led to an increase in fat deposition in the liver, resulting in vacuolar degeneration and lipid droplet accumulation in the liver [43]. Heat stress also interfered with the expression of key genes involved in fat synthesis in the liver, leading to fat deposition. Oil Red O staining results showed that the degree of lipid droplet accumulation in the livers of Peking ducks supplemented with silybin was significantly lower than that of the HS group. Therefore, it is believed that inhibition of hepatic lipid accumulation is one protective mechanism of silybin against heat stress-induced liver injury in Peking ducks.

In recent years, non-targeted metabolomics technology has been widely applied in the livestock industry. Non-targeted metabolomics technology for comprehensive, in-depth studies of differential metabolites and metabolic pathways facilitates a more intuitive understanding of the interactions between organismal metabolism and the external environment. Analysis of the significant differential metabolites revealed that triglyceride (TG) levels decreased in the HS group, which might be due to decreased feed intake observed and its resultant reduction in the amount of triglycerides obtained from food in the high-temperature environment. Similar results were also seen in other studies [44,45,46], while current data confirmed that heat-stressed Peking ducks exhibited reduced feed intake. One study found that heat stress increased the serum triglyceride concentration in broilers [11], but it is speculated that the regulatory effect of heat stress on triglycerides may be affected by specific heat stress conditions and animal species.

Sphingolipid metabolism plays an important role in regulating critical cellular and biological functions, including programmed cell death, cell growth and differentiation, and inflammatory responses. Sphingolipids are a class of cell membrane lipids with biological activities that are structurally based on sphingosine, and are also one of the key regulatory factors of metabolism [47]. Sphingomyelin and Ceramides are the two most common sphingolipids, and studies have found that endogenous ceramides or exogenous ceramide analogs promote mitochondrial autophagy and induce cancer cell death by selectively targeting signaling pathways for protection [48]. Long-chain ceramides have been identified as the predominant ceramide substances in a healthy human body, and when lipid metabolism disorder occurs, serum ceramide levels decrease [49]. In addition, ceramides can inhibit PPARγ activity, and PPARγ can directly regulate genes involved in lipid synthesis and adipocyte differentiation, thereby promoting fat accumulation [50]. Similar results were seen here, where heat stress significantly reduced the expression of ceramides in the liver, accompanied by massive lipid droplet accumulation, indicating that heat stress indeed affected normal hepatic lipid metabolism in Peking ducks by interfering with the PPARγ signaling pathway. Sphingomyelin is important for maintaining the structural homeostasis of cell membranes and also participates in regulating many biological processes in the body. Analysis showed that sphingomyelin expression increased significantly in the livers of Peking ducks fed with a diet containing silybin. It is hypothesized that silybin can maintain the structural integrity of liver cell membranes by increasing sphingomyelin expression, thereby ensuring normal cellular and hepatic metabolic activity. The glycerophospholipid metabolism pathway is a key participant in lipid metabolism, and glycerophospholipids are one of the most abundant phospholipids in the body. Changes in their synthesis and metabolism can affect glycerophospholipid content and cell membrane composition. Specifically, Phosphatidylcholine (PC) and phosphatidylethanolamine (PE) are two important glycerophospholipids and also crucial participants in lipid metabolism. Studies have found that changes in the relative proportion of PC and PE can affect the formation and size of lipid droplets, leading to lipid metabolism disorders [51]. Combined with the Oil Red O staining results, the data suggest that heat stress may have altered the proportions of various components of liver glycerophospholipids, leading to the accumulation of lipid droplets in the liver. Silybin may thus have regulated the proportions of various glycerophospholipid compounds by mediating lipid metabolic pathways, thereby partially alleviating fatty degeneration of the liver and increasing the liver’s heat tolerance of Peking ducks to environmental high temperatures.

## 5. Conclusions

Silybin partially alleviated the decreases in feed intake and body weight gain in Peking ducks caused by heat stress, although the improvements in growth-related indicators were not statistically significant. Dietary silybin, particularly at low and medium doses, protected against heat-stress-induced liver damage by enhancing liver antioxidant capacity, reducing inflammatory responses, and regulating the expression of HSPs. It also alleviated histological changes and lipid droplet accumulation in the livers of Peking ducks under heat stress, while improving liver tissue morphology and fatty degeneration. Liver lipidomic analysis showed that silybin could affect liver lipid metabolism in Peking ducks under heat stress by regulating sphingolipid and glycerophospholipid metabolic pathways. Results from this study suggest that dietary silybin, particularly at low and medium doses, may help alleviate heat-stress-induced liver damage in Peking ducks under heat-stress conditions.

## Figures and Tables

**Table 1:** Ingredients, energy content, and chemical composition of the diet (air-dry basis; %, unless otherwise stated).

Items	Day 1–14	Day 15–42
Corn	56.00	60.24
Wheat midding	5.00	9.00
Soybean meal	32.69	24.67
Soybean oil	2.10	1.80
Phytases	0.02	0.02
Dicalcium phosphate	1.00	1.60
Limestone	1.50	1.20
DL-Methionine	0.15	0.12
L-Lysine	0.20	0.10
^a^ Vitamin premix	0.02	0.02
^b^ Trace mineral premix	0.20	0.20
NaCl	0.35	0.30
Choline chloride (50%)	0.24	0.20
Ethoxyquin (33%)	0.03	0.03
Maifanite	0.50	0.50
^c^ Total	100.00	100.00
ME (MJ/kg)	12.31	12.53
Crude protein	19.52	16.83
Lysine	1.12	0.87
Methionine	0.46	0.39
Methionine + Cysteine	0.79	0.69
Calcium	0.88	0.89
Available phosphorus	0.29	0.39
Total phosphorus	0.54	0.62

^a^ Each kilogram of the vitamin premix contained: Vitamin A 12,500 IU, Vitamin D 3500 IU, Vitamin E 20 IU, Vitamin K3 2.65 mg, Vitamin B1 2.00 mg, Vitamin B2 6.00 mg, Vitamin B12 0.025 mg, Biotin 0.0325 mg, Folic Acid 12.00 mg, D-Pantothenic Acid 50.00 mg, and Niacin 50.00 mg. ^b^ Each kilogram of the trace mineral premix contained: Cu 6 mg, Zn 40 mg, Se 0.15 mg, Mn 100 mg, Fe 80 mg, and I 0.35 mg.^c^ Calculated value.

**Table 2:** Primer sequences for heat shock proteins and oxidative stress-related genes.

Genes	GenBank Accession Number	Primer Sequences (5′-3′)
*HSP60*	XM_027461761.2	F: AGAAGAAGGACAGAGTTACCGATGC
		R: ACAGCCACCACCTGGAACAATG
*HSP70*	NM_001109785.2	F: GCTCTCCTGTCCTCTGGCTTTAG
		R: GGTGGCATCTCCTCGGTAACTG
*HSF-1*	XM_027452840.2	F: ATTCCAGCACCCGTACTTCATCC
		R: GGTCAGCAGCTTGGTTACATTGTC
*Nrf2*	NM_001396902.1	F: CCACTTACTGATGCGGAGAATATGC
		R: CAGGGCTTGTGATTGTGCTTACC
*GAPDH*	XM_038180584.1	F: AGTGAAGGCTGCTGCTGATGG
		R: TCAAAGGTGGAGGAATGGCTGTC

Note: HSP60, Heat Shock Protein 60; HSP70, Heat Shock Protein 70; HSF-1, Heat Shock Factor; Nrf2, Nuclear factor erythroid 2-related factor 2; GAPDH, Glyceraldehyde-3-phosphate dehydrogenase.

**Table 3:** Statistical table of differential metabolite quantities.

	Totle			Up-Regulate			Down-Regulate		
	Total	ESI^+^	ESI^−^	Total	ESI^+^	ESI^−^	Total	ESI^+^	ESI^−^
C vs. HS	328	199	129	101	53	48	227	146	81
HS vs. L + HS	252	145	107	146	90	56	106	55	51

Note: ESI^+^, positive electrospray ionization mode; ESI^−^, negative electrospray ionization mode.

## Data Availability

The original contributions presented in this study are included in the article. Further inquiries can be directed to the corresponding authors.

## Appendix A

**Table A1:** Gradient elution program.

Time/min	Flow Rate/mL/min	Mobile Phase A/% (50% Acetonitrile Aqueous Solution)	Mobile Phase B/% (Acetonitrile/Isopropanol/Water (10/88/2)	Injection Volume/μL	Column Temperature/°C
0	0.4	65	35	5	40
4	0.4	40	60		
12	0.4	15	85		
15	0.4	0	100		
17	0.4	0	100		
18	0.4	65	35		
20	0.4	65	35		

**Table A2:** Detection parameters.

Item	Parameter
Scan Range	200–2000
Sheath Gas	60
Auxiliary Gas	20
Ion Source Heating Temperature	370
Ionization Voltage (Positive)	+3000
Ionization Voltage (Negative)	−3000
Collision Energy	20–4–60

**Table A3:** Evaluation parameters of the OPLS-DA model.

	Type	R2X	R2Y	Q2
C vs. HS	ESI^+^	0.586	0.994	0.825
	ESI^−^	0.418	0.987	0.725
HS vs. L + HS	ESI^+^	0.514	0.992	0.743
	ESI^−^	0.537	0.998	0.797

**Table A4:** Top 30 significantly different metabolites in VIP ranking between C and HS groups.

Lipid	Class Name	VIP	*p* Value	Regulate
MGDG (32:1/20:4)	Monogalactosyldiacylglycerol	3.5463	0.02082	up
TG (30:1/18:1/18:2)	triglyceride	2.9864	0.0172	down
TG (16:0/10:0/22:5)	triglyceride	2.8526	0.02207	up
TG (22:4/20:4/22:5)	triglyceride	2.7483	0.02202	down
TG (18:1/17:1/22:6)	triglyceride	2.6715	0.03097	down
TG (26:0/16:0/18:1)	triglyceride	2.6059	0.02195	down
OAHFA (20:3/20:2)	OAcyl-(gamma-hydroxy) FA	2.6001	0.004442	down
TG (18:1e/18:1/22:5)	triglyceride	2.5799	0.03102	down
LPE (20:3)	Lysophosphatidylethanolamine	2.5128	0.0002085	down
LPE (20:4)	Lysophosphatidylethanolamine	2.4837	0.000007259	down
PI (16:0/20:3)	phosphatidylinositol	2.4227	0.00001378	up
OAHFA (22:4/22:3)	OAcyl-(gamma-hydroxy) FA	2.4078	0.0001611	down
TG (18:1e/18:1/18:2)	triglyceride	2.4036	0.008352	down
TG (18:0/17:0/18:2)	triglyceride	2.4015	0.0001672	up
TG (20:1e/18:1/22:4)	triglyceride	2.3986	0.02596	down
Cer (t18:1/23:1)	Ceramides	2.3656	0.002675	down
TG (19:1/18:1/18:1)	triglyceride	2.3289	0.01012	down
TG (20:0e/18:0/18:1)	triglyceride	2.3144	0.006078	down
Cer (t18:1/22:1)	Ceramides	2.3126	0.003971	down
DG (22:5/20:4)	diglyceride	2.2814	0.000809	down
LPE (20:5)	lysophosphatidylethanolamine	2.2699	0.007622	down
TG (17:0/20:4/20:4)	triglyceride	2.2408	0.004988	down
TG (20:4/20:4/20:4)	triglyceride	2.2199	0.0006105	down
BisMePA (8:1e/10:4)	Bis-methyl phosphatidic acid	2.2141	0.000002655	down
PI (20:4/20:4)	phosphatidylinositol	2.1728	0.0004345	down
PC (16:1/23:0)	phosphatidylcholine	2.1685	0.02884	down
Cer (d24:1/18:1 + O)	Ceramides	2.1518	0.004475	down
Cer (d23:1/18:1 + O)	Ceramides	2.1518	0.004475	down
TG (20:0e/18:1/18:1)	triglyceride	2.1482	0.002146	down
DG (38:1/18:1)	diglyceride	2.1482	0.002146	down

**Table A5:** Top 30 significantly different metabolites between HS and L + HS groups.

Lipid	Class Name	VIP	*p* Value	Regulate
SM (d18:0/16:0)	sphingomyelin	3.8879	0.002691	up
TG (18:2/18:2/23:0)	triglyceride	3.4648	0.0005734	up
SM (d17:0/18:1)	sphingomyelin	3.4538	0.004179	up
SM (d18:1/18:0)	sphingomyelin	3.3922	0.006515	up
SM (d17:1/16:0)	sphingomyelin	3.3802	0.01379	up
SM (t18:1/16:0)	sphingomyelin	3.3497	0.00389	up
CL (23:0/16:0/16:0/18:1)	Cardiolipin	3.1174	0.02184	up
SM (d18:1/20:0)	sphingomyelin	3.104	0.005797	up
OAHFA (20:4/20:4)	OAcyl-(gamma-hydroxy) FA	3.0258	0.0464	up
SM (d19:0/18:1)	sphingomyelin	3.021	0.002829	up
SM (d18:1/18:3)	sphingomyelin	3.0004	0.02162	up
SM (d19:0/24:1)	sphingomyelin	2.8861	0.002841	up
SM (d18:1/21:0)	sphingomyelin	2.8729	0.007409	up
SM (t18:1/22:0)	sphingomyelin	2.849	0.001196	up
SM (d20:0/18:1)	sphingomyelin	2.8246	0.01815	up
SM (d18:0/18:0)	sphingomyelin	2.8195	0.009318	up
DG (20:4/22:6)	diglyceride	2.8149	0.0001331	up
SM (d14:0/18:1)	sphingomyelin	2.8092	0.04543	up
MGDG (20:2e/18:0)	Monogalactosyldiacylglycerol	2.7635	0.009121	up
SM (t18:1/23:0)	sphingomyelin	2.7585	0.002559	up
SM (d18:1/22:0)	sphingomyelin	2.7481	0.005023	up
SM (d18:1/16:0)	sphingomyelin	2.7429	0.01262	up
SM (t18:1/24:0)	sphingomyelin	2.708	0.003679	up
TG (30:0/18:1/22:4)	triglyceride	2.6968	0.03175	down
SM (d18:1/24:2)	sphingomyelin	2.6451	0.01091	up
OAHFA (18:2/20:4)	OAcyl-(gamma-hydroxy) FA	2.6267	0.02437	up
SM (d18:1/24:3)	sphingomyelin	2.5826	0.008192	up
SM (d18:1/22:2)	sphingomyelin	2.5818	0.0001279	up
Hex1SPH (d18:0)	Glucosylsphingosine	2.5747	0.002535	down
SM (d18:1/24:0)	sphingomyelin	2.5638	0.01007	up

**Table A6:** Important lipid metabolic pathways.

Pathway Description	Pathway ID	Match Status	Impact
Sphingolipid metabolism	map00600html	1|33; 2|33	0.223480334; 0.223480334
Glycerophospholipid metabolism	map00564html	2|52; 4|52	0.063866381; 0.150537008
Glycerolipid metabolism	map00561html	1|32; 1|32	0.005301524; 0.005301524
Linoleic acid metabolism	map00591html	1|13; 1|13	<0.001; <0.001
alpha-Linolenic acid metabolism	map00592html	1|30; 1|30	<0.001; <0.001
Arachidonic acid metabolism	map00590html	1|46; 1|46	<0.001; <0.001

Note: In the “Match status” and “Impact” columns, the data on the left side represent the comparison results between the C group and the HS group, while the data on the right side represent the comparison results between the HS group and the L + HS group.
